# Elevated n-3/n-6 PUFA ratio in early life diet reverses adverse intrauterine kidney programming in female rats

**DOI:** 10.1016/j.jlr.2022.100283

**Published:** 2022-09-21

**Authors:** Jenny Voggel, Gregor Fink, Magdalena Zelck, Maria Wohlfarth, Julia M. Post, Laura Bindila, Manfred Rauh, Kerstin Amann, Miguel A. Alejandre Alcázar, Jörg Dötsch, Kai-Dietrich Nüsken, Eva Nüsken

**Affiliations:** 1 Clinic and Polyclinic for Pediatric and Adolescent Medicine, Faculty of Medicine and University Hospital Cologne, University of Cologne, Cologne, Germany; 2 Center for Molecular Medicine Cologne (CMMC), University of Cologne, Cologne, Germany; 3 Clinical Lipidomics Unit, Institute of Physiological Chemistry, University Medical Center of the Johannes Gutenberg University of Mainz, Mainz, Germany; 4 Department of Pediatrics and Adolescent Medicine, University Hospital Erlangen, Erlangen, Germany; 5 Department of Nephropathology, Institute of Pathology, Friedrich-Alexander-University Erlangen, Erlangen, Germany; 6 Cologne Excellence Cluster on Cellular Stress Responses in Aging-Associated Diseases (CECAD), University of Cologne, Cologne, Germany; 7 Institute for Lung Health, German Center for Lung Research (DZL), University of Giessen and Marburg Lung Center (UGMLC), Gießen, Germany

**Keywords:** arachidonic acid, developmental origins of health and disease, eicosanoids, chronic kidney disease, lipidomics, nutrition, omega-3 fatty acids, perinatal programming, placental insufficiency, proteomics, AA, arachidonic acid, C, control group, CKD, chronic kidney disease, CONTR, control diet, DiHETrE, dihydroxyeicosatrienoic acid, EET, epoxyeicosatrienoic acid, fc, fold change, GD, gestational day, GO, Gene Ontology, IUGR, intrauterine growth restriction, IUS, intrauterine stress, LA, linoleic acid, LIG, ligation, LPC, lyso-phosphatidylcholine, LSR, lipolysis-stimulated lipoprotein receptor, MRM, multiple reaction monitoring, P, postnatal, PE, phosphatidylethanolamine, PGE_2_, prostaglandin E_2_, PL, phospholipid, PLA_2_, phospholipase A_2_, TMA, thrombotic microangiopathy

## Abstract

Intrauterine growth restriction (IUGR) predisposes to chronic kidney disease via activation of proinflammatory pathways, and omega-3 PUFAs (n-3 PUFAs) have anti-inflammatory properties. In female rats, we investigated *1*) how an elevated dietary n-3/n-6 PUFA ratio (1:1) during postnatal kidney development modifies kidney phospholipid (PL) and arachidonic acid (AA) metabolite content and *2*) whether the diet counteracts adverse molecular protein signatures expected in IUGR kidneys. IUGR was induced by bilateral uterine vessel ligation or intrauterine stress through sham operation 3.5 days before term. Control (C) offspring were born after uncompromised pregnancy. On postnatal (P) days P2–P39, rats were fed control (n-3/n-6 PUFA ratio 1:20) or n-3 PUFA intervention diet (N3PUFA; ratio 1:1). Plasma parameters (P33), kidney cortex lipidomics and proteomics, as well as histology (P39) were studied. We found that the intervention diet tripled PL-DHA content (PC 40:6; *P* < 0.01) and lowered both PL-AA content (PC 38:4 and lyso-phosphatidylcholine 20:4; *P* < 0.05) and AA metabolites (HETEs, dihydroxyeicosatrienoic acids, and epoxyeicosatrienoic acids) to 25% in all offspring groups. After ligation, our network analysis of differentially expressed proteins identified an adverse molecular signature indicating inflammation and hypercoagulability. N3PUFA diet reversed 61 protein alterations (*P* < 0.05), thus mitigating adverse IUGR signatures. In conclusion, an elevated n-3/n-6 PUFA ratio in early diet strongly reduces proinflammatory PLs and mediators while increasing DHA-containing PLs regardless of prior intrauterine conditions. Counteracting a proinflammatory hypercoagulable protein signature in young adult IUGR individuals through early diet intervention may be a feasible strategy to prevent developmentally programmed kidney damage in later life.

## Graphical abstract

**Figure:**
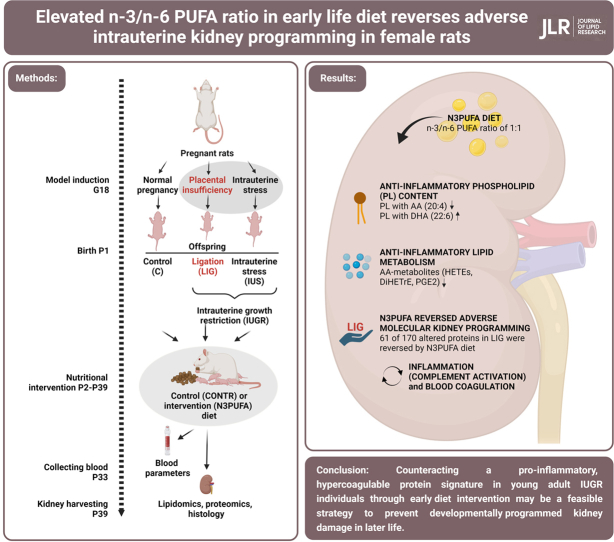

Intrauterine growth restriction (IUGR) describes a condition in which affected fetuses do not reach their predicted growth potential. It is a major cause of low birth weight, which occurs in around 15% of newborns worldwide (1). Epidemiological studies have demonstrated a correlation between IUGR and impaired kidney function in early adulthood (2), elevated blood pressure (3), an adverse course of glomerulopathies (4, 5), and an overall elevated risk of end-stage renal disease (6, 7). Consequently, IUGR is a substantial determinant for kidney health throughout life. Placental insufficiency and intrauterine stress (IUS) are common causes of IUGR (8) and have been comprehensively studied in animal models to elucidate the morphological, physiological, and molecular consequences (9, 10, 11, 12). However, strategies to protect vulnerable IUGR-born offspring from adverse kidney outcome are still scarce.

In the context of kidney disease, accumulating evidence shows that arachidonic acid (AA)-derived lipid mediators are involved in the pathogenesis of arterial hypertension and chronic kidney disease (CKD) (13), whereas n-3 PUFAs have renoprotective properties under certain conditions. It is well known that supplementation of EPA and DHA can be beneficial in cardiovascular (14) and inflammatory diseases (15). Specifically, it has been shown that PUFA supplementation augments the benefits of renin-angiotensin-aldosterone system inhibition in IgA nephritis (16). In diabetic nephropathy, diet interventions targeting n-3 PUFAs have been successful in ameliorating proteinuria (17). From a molecular view, it has been suggested that anti-inflammatory and antihypertensive effects are mediated by increased concentrations of n-3 PUFAs in membrane phospholipids (PLs) (18) and by a shift in AA-derived vasoactive and immune-modulating eicosanoids (19).

Of note, adverse molecular signatures in IUGR kidneys can be found well before the onset of clinical or morphological alterations (20). This suggests the existence of a “window of opportunity,” during which susceptibility toward kidney disease can be reversed in IUGR individuals. Based on prior observations that *1*) IUGR predisposes to CKD via proinflammatory pathways (20, 21) and *2*) n-3 PUFAs have anti-inflammatory properties (15, 17), we hypothesized that a lipid-modified dietary intervention during postnatal kidney development could mitigate vulnerability toward apparent kidney disease. In detail, we investigated in female rats *1*) how an elevated dietary n-3/n-6 PUFA ratio (1:1) during postnatal rat kidney development modifies kidney PL and AA metabolite content and *2*) whether the diet counteracts adverse molecular protein signatures expected in IUGR kidneys.

## Materials and methods

### Animal model and in vivo studies

Experimental IUGR was induced through placental insufficiency by bilateral uterine vessel ligation (LIG) or IUS through “sham operation” of rat dams on gestational day (GD) 18 (i.e., 3.5 days before term). These models were chosen because they have been widely used to simulate the respective human conditions (11, 12, 20, 22, 23). All animal procedures were in accordance with German regulations and legal requirements. The appropriate Institutional and Governmental Review Boards approved the experimental protocol (LANUV NRW AZ 81-02.04.2018.A052).

In detail, Wistar rat dams were time mated (the presence of sperm plug = GD0). On GD18, LIG dams were narcotized, and the uterine arteries and veins were bilaterally ligated. IUS dams underwent a “sham operation” as described previously (12, 20, 22). All rat offspring were born spontaneously within 12 h around GD21.5 (≙postnatal [P] day 1). While giving birth, the dams and respective original litters were not disturbed to avoid stress. On P2, original litters with 9–16 pups were characterized by sex, birth length, and birth weight. To limit adverse environmental conditions in IUGR offspring to the prenatal period, all newborn rats were fostered to unimpaired C mothers. Foster litters were composed of six male and two female offspring to avoid confounding by abnormal sex ratio. Pups within a foster litter were taken from four separate original litters (two pairs of male siblings were selected from two original litters, from further two original litters, we chose one male and one female sibling, respectively). In total, each experimental group was represented by eight original litters (C-CONTR, C-N3PUFA, LIG-CONTR, LIG-N3PUFA, IUS-CONTR, and IUS-N3PUFA). C and IUS offspring were randomly selected for fostering, whereas LIG offspring were randomly selected from rats with a birth weight below the 10th percentile, based on the weights of C offspring and corresponding to the human small for gestational age definition (24). The selection was necessary to exclude offspring after insignificant uteroplacental insufficiency, which is physiologically present in the uterine positions far from the LIG and causes less severe IUGR and associated metabolic sequels (22, 23). All dams were fed control diet (CONTR) C1000 modified #100228 (Altromin, Lage, Germany), containing 1 g choline chloride/kg food and an n-3/n-6 ratio of 1:20 until giving birth. Starting on P2, half of the foster dams and the respective offspring remained on the CONTR diet, whereas the other half was fed n-3 PUFA intervention diet (N3PUFA). N3PUFA diet consisted of a mixture of C1000 modified #100230 diet (Altromin) and Incromega™ DHA 500TG (Corda, Nettetal, Germany) leading to 5 g choline chloride/kg food, 5 g DHA/kg food, and an n-3/n-6 ratio of 1:1. To limit chemical alterations of the diet ingredients by oxygen and temperature, the diet was freshly mixed every 2–3 days in our laboratory. All ingredients of both diets are listed in Table 1. Offspring was weaned on P21. On P33, rats were fasted for 6 h, and retro-orbital blood was collected. On P39, cardiac perfusion was performed in female rats with PBS at room temperature. Kidneys were collected and prepared for molecular and histological analyses. All male rats were kept within the study for further research questions.

**Table 1:** Ingredients of the diets

Ingredients (mg/kg, ^Δ^kcal/kg, ∗IE/kg)	Control diet (CONTR)	Intervention diet (N3PUFA)	Ratio N3PUFA/CONTR
Metabolic energy^Δ^	3,506	3,501	1.00
Crude protein	172,850	172,614	1.00
Crude fat	49,829	52,940	1.06
Polysaccharide(s)	471,727	465,292	0.99
Disaccharide(s)	98,105	98,105	1.00
Crude fiber	30,970	30,948	1.00
Alanine	2,528	2,525	1.00
Arginine	9,829	9,827	1.00
Aspartic acid	3,583	3,581	1.00
Cysteine	3,196	3,195	1.00
Glutamic acid	23,675	23,668	1.00
Glycine	3,136	3,135	1.00
Histidine	5,276	5,275	1.00
Isoleucine	7,223	7,221	1.00
Leucine	14,763	14,758	1.00
Lysine	17,401	17,400	1.00
Methionine	7,223	7,222	1.00
Phenylalanine	7,172	7,170	1.00
Proline	12,763	12,760	1.00
Serine	5,268	5,266	1.00
Threonine	7,154	7,153	1.00
Tryptophan	1,977	1,977	1.00
Tyrosine	9,285	9,283	1.00
Valine	3,296	3,294	1.00
Aluminium	4	4	1.00
Biotin	0.2	0.2	1.00
Calcium	9,311	9,305	1.00
Chlorine	3,630	3,630	1.00
Cobalt	0.1	0.1	1.00
Copper	6	6	1.00
Digested phosphorus	7,200	7,199	1.00
Fluorine	4	4	1.00
Folic acid	10	10	1.00
Iodine	0.5	0.5	1.00
Iron	179	179	1.00
Magnesium	684	683	1.00
Manganese	101	101	1.00
Molybdenum	0.2	0.2	1.00
Nicotinic acid	50	50	1.00
Pantothenic acid	50	50	1.00
Potassium	7,089	7,088	1.00
Selenium	0.3	0.3	1.00
Sodium	2,488	2,487	1.00
Sulfur	2,792	2,790	1.00
Vitamin A∗	15,000	15,000	1.00
Vitamin B1	20	20	1.00
Vitamin B12	0.04	0.04	1.00
Vitamin B2	20	20	1.00
Vitamin B6	15	15	1.00
Vitamin C	20	20	1.00
Vitamin D3∗	500	500	1.00
Vitamin E	163	160	0.98
Vitamin K3 as menadione	10	10	1.00
Zinc	29	29	1.00
Choline chloride	1,012	5,002	4.94
Capric acid 10:0	30	0	0.00↓
Lauric acid 12:0	30	35	1.17↑
Myristoleic acid 14:0	30	2,323	77.43↑
Pentadecanoic acid 15:0	30	141	4.70↑
Palmitic acid 16:0	8,662	6,229	0.72↓
Palmitoleic acid 16:1	30	3,238	107.93↑
Margaric acid 17:0	30	0	0.00↓
Stearic acid 18:0	7,326	1,407	0.19↓
Oleic acid 18:1	13,732	6,120	0.45↓
LA 18:2n-6	17,000	10,995	0.65↓
Linolenic acid 18:3n-3	809	633	0.78↓
Arachidic acid 20:0	270	429	1.59↑
Eicosenoic acid 20:1	360	739	2.05↑
Eicosadienoic acid 20:2	270	35	0.13↓
AA 20:4n-6	30	551	18.37↑
EPA 20:5n-3	30	7,562	252.07↑
Behenic acid 22:0	180	35	0.19↓
Erucic acid 22:1	0	986	—↑
Docosapentaenoic acid 22:5n-3	0	186	—↑
Docosapentaenoic acid 22:5n-6	0	373	—↑
DHA 22:6n-3	30	5,000	166.67↑
Nervonic acid 24:1	30	0	0.00↓
n-3/n-6 fatty acid ratio	1:20	1:1	—

Altromin provided both diets. All dams were fed control diet (CONTR) C1000 modified #100228 (Altromin) until giving birth. Starting on P2, half of the foster dams and the respective offspring remained on the CONTR diet, whereas the other half was fed n-3 PUFA intervention diet (N3PUFA). The intervention diet was a mixture of the C1000 modified #100230 diet (Altromin) and Incromega™ DHA 500TG (Croda GmbH). This mixture was self-made.

### Laboratory measurement of blood parameters

Blood plasma parameters were measured with established methods at the Institute of Clinical Chemistry of the University Hospital Cologne and at the Clinical Laboratory at the Department of Pediatrics at the University Hospital of Erlangen. Plasma samples were centrifuged at 2,000 *g* for 5 min at 4°C. Creatinine, triglycerides, and cholesterol were measured by an enzymatic colorimetric assay and HDL by a homogeneous enzymatic colorimetric assay from Roche Diagnostics. For detailed assay information, please see supplemental Table 1.

### Histology

Slices were stained with H&E for standard morphological characterization or with Picro-Sirius Red for collagen assessment. First, 3 μm kidney middle sections were deparaffinized by default. For H&E, slices were stained in Mayer's hematoxylin for 4 min and in eosin for 2 min and mounted in NeoMount after dehydration. For Picro-Sirius Red, slices were pretreated with 0.2% phosphomolybdic acid solution for 3 min, followed by 1 h incubation with the Picro-Sirius Red solution. After staining, samples were dipped in 0.5% acetic acid solution, 100% ethanol, and Neo-Clear before being mounted in NeoMount. The cortex-medulla region was selected, as it is the functional most relevant region and clearly separable from the pelvis region. Areas stained by Sirius Red were quantified using a threshold-based positive pixel count procedure of the QuPath software (version 0.2.3) (25). Areas stained “positive” were divided by the region of interest.

For immunohistochemical stainings, slides were deparaffinized and boiled at 90°C for 20 min in a citrate buffer (pH = 6) for heat-induced epitope retrieval. Tissue was treated in 3% H_2_O_2_ in distilled water for 10 min, followed by PBS with 0.01% Tween-20. Blocking was performed using 5% goat serum with 300 mM glycine in PBS for 1.5 h followed by primary antibody incubation (CD68 [Abcam, catalog no.: ab31630], 1:500 dilution; WT-1 [Abcam, catalog no.: ab212951], 1:800 dilution; and Ki67 [Thermo Fisher Scientific, catalog no.: 14-598-82], 1:500 dilution) overnight at 4°C. The next day, slides were washed with PBS and incubated with a secondary antibody conjugated with horseradish peroxidase (ZytoChem Plus HRP One-Step Polymer) for 30 min. Slices were treated with 3-amino-9-ethylcarbazole until a rich positive red staining was noticeable using a light microscope. Consecutively, nuclei were stained in hematoxylin solution, and sections were dehydrated and mounted with Neo Mount. Stainings were quantified using a threshold-based positive pixel count procedure of the QuPath software (version 0.2.3) (25). Cells stained “positive” were divided by the region of interest.

### Kidney cortex lipidomics

Lipid extraction and lipidomic analyses were performed using established methods as published before (26). Because of financial and practical reasons, we had to limit the number of metabolites. PLs that are known to be relevantly expressed in kidney tissue were measured. However, fatty acids with C <10 and >22 were not measured, since they were not ingredients of our diets and are not known to be quantitatively relevant in kidney cortex tissue. Selection of eicosanoids was guided by the expected influence on biosynthesis steps following a dietary n3-PUFA intervention. In addition, we focused on parameters with well-known relevance in proinflammatory and anti-inflammatory processes. For this purpose, literature mining on molecular players in kidney cortex following dietary intervention was applied to select the most relevant and interesting HETEs, dihydroxyeicosatrienoic acid (DiHETrE), epoxyeicosatrienoic acid (EETs), and prostaglandin E_2_ (PGE_2_) (27, 28).

Briefly, kidney cortex tissue samples (P39) for both PL and eicosanoid analysis were spiked with internal standards (supplemental Table 2). The internal standards were used according to Post *et al.* and Lerner *et al.* (26, 29) and subsequently extracted using methyl-tert-butyl-ether-based liquid-liquid extraction protocols for PLs and eicosanoids, respectively (30). Selected PLs and eicosanoid species were analyzed using liquid-chromatography multiple reaction monitoring (MRM) on a QTRAP 5500 (AB Sciex, Darmstadt, Germany) operating in positive negative ion mode switching, using analytical conditions described (26, 29). For targeted quantification, MRM transitions of the molecular ions to class-specific ion fragments were used. For PL analysis also to fatty acyl chain ion fragments to dissect the peaks corresponding to the targeted PL composition. Aqueous phase resulting from liquid-liquid extraction lipid extraction was used for protein content determination of the tissues using a BCA assay (FLUOstar instrument) (26, 31). The lipid values determined by LC/MRM were normalized to tissue protein content. LC/MRM data were processed using MultiQuant 3.03 (AB Sciex).

**Table 2:** Metabolic parameters in blood plasma on P33

Metabolic parameter	C-CONTR	C-N3PUFA	LIG-CONTR	LIG-N3PUFA	IUS-CONTR	IUS-N3PUFA^†^	Significances, *P* < 0.05
Triglycerides (mg/dl)	100.3 ± 25.95	71.98 ± 14.37	83.03 ± 16.10	68.92 ± 17.12	112.4 ± 31.09	**53.32 ± 13.80**	†
Cholesterol (mg/dl)	97.08 ± 14.45	88.38 ± 10.11	103.7 ± 13.88	90.90 ± 8.90	93.52 ± 14.15	85.76 ± 7.91	—
HDL (mg/dl)	71.67 ± 11.26	61.43 ± 6.88	77.60 ± 10.85	67.14 ± 7.77	69.54 ± 11.94	62.84 ± 5.91	—
Alanine transaminase (U/I)	30.45 ± 5.87	22.58 ± 15.67	20.43 ± 11.15	13.45 ± 13.36	21.57 ± 9.79	29.34 ± 13.04	—
Aspartate transaminase (U/I)	89.64 ± 5.46	85.00 ± 10.06	92.82 ± 8.32	86.10 ± 5.02	91.76 ± 10.25	86.50 ± 19.59	—
Cystatin C (mg/l)	0.79 ± 0.14	0.81 ± 0.21	0.83 ± 0.13	0.73 ± 0.09	0.77 ± 0.10	**1.07 ± 0.40**	†
Total protein (g/l)	53.66 ± 1.55	53.77 ± 1.24	53.04 ± 1.52	54.54 ± 1.06	54.4 ± 1.88	53.56 ± 1.69	—
Creatinine (mg/dl)	0.24 ± 0.10	0.20 ± 0.03	0.20 ± 0.03	0.19 ± 0.01	0.21 ± 0.02	0.20 ± 0.02	—
Albumin (g/l)	38.1 ± 1.37	38.47 ± 1.79	37.66 ± 1.63	38.79 ± 1.15	38.35 ± 2.02	38.37 ± 1.3	—
Urea (mg/l)	21.56 ± 5.72	19.95 ± 6.19	21.29 ± 1.97	23.4 ± 4.55	26.55 ± 6.85	**16.77 ± 5.25**	†
Calcium (mmol/l)	2.71 ± 0.06	2.73 ± 0.05	2.46 ± 0.72	2.74 ± 0.03	2.71 ± 0.06	2.68 ± 0.02	—
Phosphate (mmol/l)	2.74 ± 0.23	2.69 ± 0.24	2.57 ± 0.08	2.58 ± 0.13	2.8 ± 0.20	2.58 ± 0.15	—
Natrium (mg/l)	137.5 ± 1.86	138 ± 1.41	137.9 ± 0.90	137 ± 1.00	137 ± 1.51	138.4 ± 1.19	—
Potassium (mg/l)	4.32 ± 0.14	4.54 ± 0.50	4.52 ± 0.21	4.25 ± 0.16	4.44 ± 0.12	4.25 ± 0.21	—

Blood metabolic parameters of female rats during the phase of the diet intervention on P33. Values are shown from *n* = 8 rats per group and represented as mean ± SD. A Mann-Whitney test was performed between each of the group comparisons, and further, Bonferroni correction was applied. Statistically significant values appear bold. Significance was considered as the adjusted *P* value of *P* < 0.05. Significant differences between groups were marked with symbols (^†^IUS-N3PUFA vs. IUS-CONTR).

**Table 3:** Lipidomics of cortex tissue on P39 with parameters sorted by diet induced fc; PLs, upper half; AA metabolites, lower half

Lipidomics, P39 (nmol/g)	C-CONTR	C-N3PUFA^#^	LIG-CONTR^§^	LIG-N3PUFA^Δ^	IUS-CONTR	IUS-N3PUFA^†^	Significances, *P* < 0.05	Regulation by N3PUFA diet
PLs								
PC 38:4 (18:0/20:4)	95557.83 ± 39734.69	**26089.05 ± 7063.86**	79321.99 ± 20867.38	**22419.12 ± 5761.88**	71262.33 ± 14097.72	**25468.08 ± 8225.90**	#, Δ, †	↓
LPC 20:4	906.63 ± 398.07	**315.02 ± 54.46**	767.35 ± 220.53	**286.15 ± 80.30**	784.81 ± 122.47	**289.02 ± 76.62**	#, Δ, †	↓
PE 38:4 (18:0/20:4)	31221.95 ± 7515.15	**20867.57 ± 2147.72**	27376.06 ± 4970.50	19883.14 ± 4273.52	26274.53 ± 4097.79	20243.41 ± 6447.32	#	↓
PC 38:5 (18:1/20:4)	29529.32 ± 12016.48	**14763.09 ± 3128.35**	25132.74 ± 6469.88	12985.29 ± 1597.77	21926.24 ± 6382.9	14510.12 ± 3298.17	#	↓
PE 36:1 (18:0/18:1)	2604.23 ± 1015.24	**4718.93 ± 951.83**	1939.07 ± 581.46	**3835.07 ± 716.10**	2123.18 ± 409.86	**4345.43 ± 1384.61**	#, Δ, †	↑
PE 34:1 (16:0/18:1)	4192.17 ± 1765.03	7001.89 ± 1063.99	3295.1 ± 942.88	**6428.67 ± 985.96**	3447.55 ± 662.07	**7355.56 ± 1925.93**	Δ, †	↑
PC 36:2 (18:1/18:1)	23143.7 ± 11467.73	30459.74 ± 6743.23	18090.87 ± 4767.17	**28932.27 ± 6102.65**	19370.79 ± 5409.55	31548.42 ± 10531.45	Δ	↑
LPE 18:0	1566.53 ± 579.29	1914.78 ± 532.83	1302.4 ± 350.69	1765.81 ± 426.13	1183.49 ± 265.97	**2131.85 ± 465.08**	†	↑
LPC 18:1	518.68 ± 191.90	596.06 ± 93.78	424.08 ± 121.30	567.82 ± 86.79	444.74 ± 102.35	**628.26 ± 141.07**	†	↑
PE 40:6 (18:0/22:6)	353.12 ± 93.55	**1426.99 ± 274.45**	302.43 ± 80.35	**1398.1 ± 286.13**	255.44 ± 53.95	**1397.52 ± 395.71**	#, Δ, †	↑
PC 40:6 (18:0/22:6)	4353.83 ± 1188.40	**11596.89 ± 1684.43**	4229.62 ± 919.68	**11765.45 ± 2299.84**	3423.19 ± 1087.47	**13319.29 ± 2822.37**	#, Δ, †	↑
SM (d18:1/24:0)	58266.50 ± 10130.57	51502.42 ± 8767.76	**21707.73 ± 7839.52**	30836.28 ± 14941.28	40032.35 ± 13240.96	34236.21 ± 18745.09	§	=
AA metabolites								
FA 20:4	2,427 ± 510.40	**946.88 ± 211.81**	2938.17 ± 427.74	**990.54 ± 234.34**	2407.22 ± 702.38	**1185.22 ± 296.49**	#, Δ, †	↓
8,9-DiHETrE	3.41 ± 1.43	**0.66 ± 0.28**	2.56 ± 1.58	1.00 ± 0.44	3.38 ± 1.47	**0.86 ± 0.57**	#, †	↓
12(S)-HETE	3.67 ± 2.72	1.01 ± 0.53	7.92 ± 6.30	0.88 ± 0.37	4.85 ± 3.84	**1.20 ± 0.88**	†	↓
11,12-EET	17.44 ± 4.84	**7.27 ± 1.22**	23.97 ± 8.39	**9.33 ± 1.45**	20.44 ± 6.02	**8.08 ± 3.55**	#, Δ, †	↓
14,15-DiHETrE	2.69 ± 1.15	**0.80 ± 0.41**	2.40 ± 1.54	1.00 ± 0.23	2.44 ± 0.67	**1.00 ± 0.67**	#, †	↓
11,12-DiHETrE	9.92 ± 4.03	**2.26 ± 1.02**	7.75 ± 5.39	2.51 ± 0.63	9.49 ± 4.41	2.61 ± 1.99	#	↓
8(S)-HETE	0.62 ± 0.21	**0.20 ± 0.05**	0.90 ± 0.36	**0.24 ± 0.10**	0.63 ± 0.30	**0.16 ± 0.07**	#, Δ, †	↓
5(S)-HETE	0.82 ± 0.25	**0.38 ± 0.08**	1.00 ± 0.34	**0.40 ± 0.07**	1.08 ± 0.31	**0.39 ± 0.24**	#, Δ, †	↓
14,15-EET	1.24 ± 0.51	**0.39 ± 0.20**	1.64 ± 0.43	**0.46 ± 0.19**	1.12 ± 0.95	0.46 ± 0.28	#, Δ	↓
PGE_2_	1.10 ± 0.54	**0.27 ± 0.10**	1.33 ± 1.15	**0.31 ± 0.15**	0.92 ± 0.24	0.40 ± 0.38	#, Δ	↓
15(S)-HETE	1.76 ± 0.56	**0.68 ± 0.31**	1.98 ± 0.59	**0.84 ± 0.26**	1.76 ± 0.82	0.74 ± 0.30	#, Δ	↓
20-HETE	2.88 ± 1.11	**0.94 ± 0.43**	4.09 ± 2.68	1.25 ± 0.38	3.73 ± 2.01	1.03 ± 0.60	#	↓

PLs and AA metabolites from female kidney cortex tissue after the diet intervention on P39. PLs were sorted against their fc between C-N3PUFA and C-CONTR within groups *1*) of PLs with AA (20:4), *2*) with saturated fatty acids or monounsaturated fatty acids, *3*) with DHA (22:6), and *4*) SM. AA metabolites were sorted against their fc in C-N3PUFA versus C-CONTR. Values are shown from *n* = 6 rats per group and represent as means ± SD. A Mann-Whitney test was performed between each of the group comparisons, and further, Bonferroni correction was applied. Statistically significant values appear bold. Significance was considered as the adjusted *P* value of *P* < 0.05. PLs and lipid mediators are only shown if there were significant differences in at least one group. Significant differences were marked with symbols (^#^C-N3PUFA vs. C-CONTR; ^Δ^LIG-N3PUFA vs. LIG-CONTR; ^†^IUS-N3PUFA vs. IUS-CONTR; and ^§^LIG-CONTR vs. C-CONTR). Corresponding heat maps are shown in Figs. 3, 4.

**Fig. 1:**
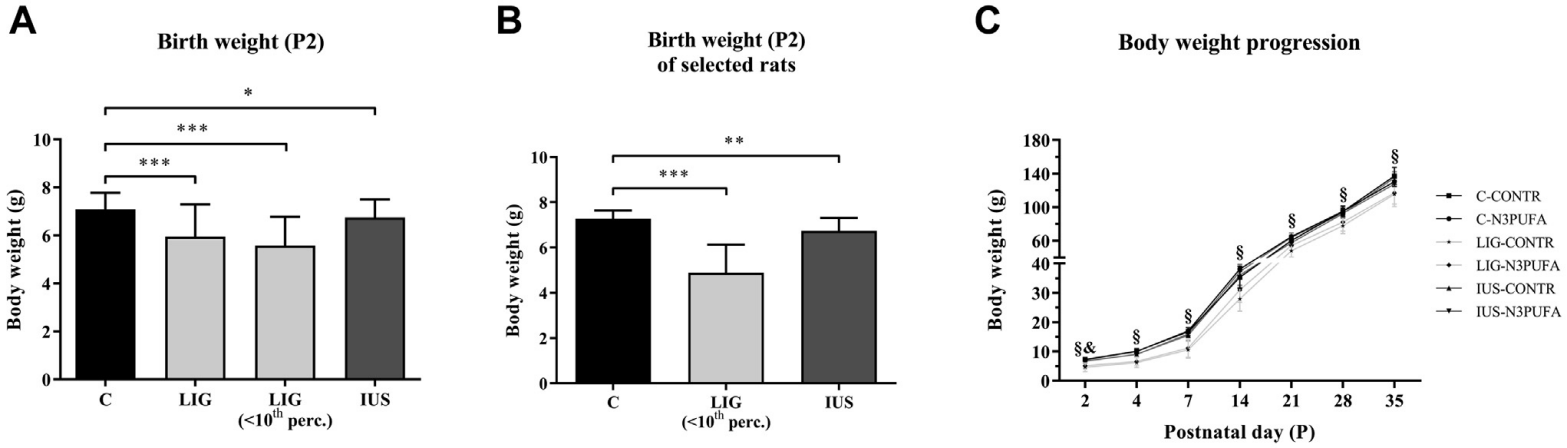
Body weights of female rats. A: Birth weights on postnatal day (P) 2; C, *n* = 98; LIG, *n* = 49; LIG (<10th percentile), *n* = 39; IUS, *n* = 67 rats per group. B: Birth weights of selected rats on P2 included in the study; C, *n* = 17; LIG (<10th percentile), *n* = 18; IUS, *n* = 11. C: Body weight progression between P2 and P35, “§” indicates significant differences (*P* < 0.05) between LIG-CONTR and C-CONTR, “&” between IUS-CONTR and C-CONTR. Mean ± SD; Bonferroni-adjusted Mann-Whitney test, ∗*P* < 0.05, ∗∗*P* < 0.01, and ∗∗∗*P* < 0.001.

**Fig. 2:**
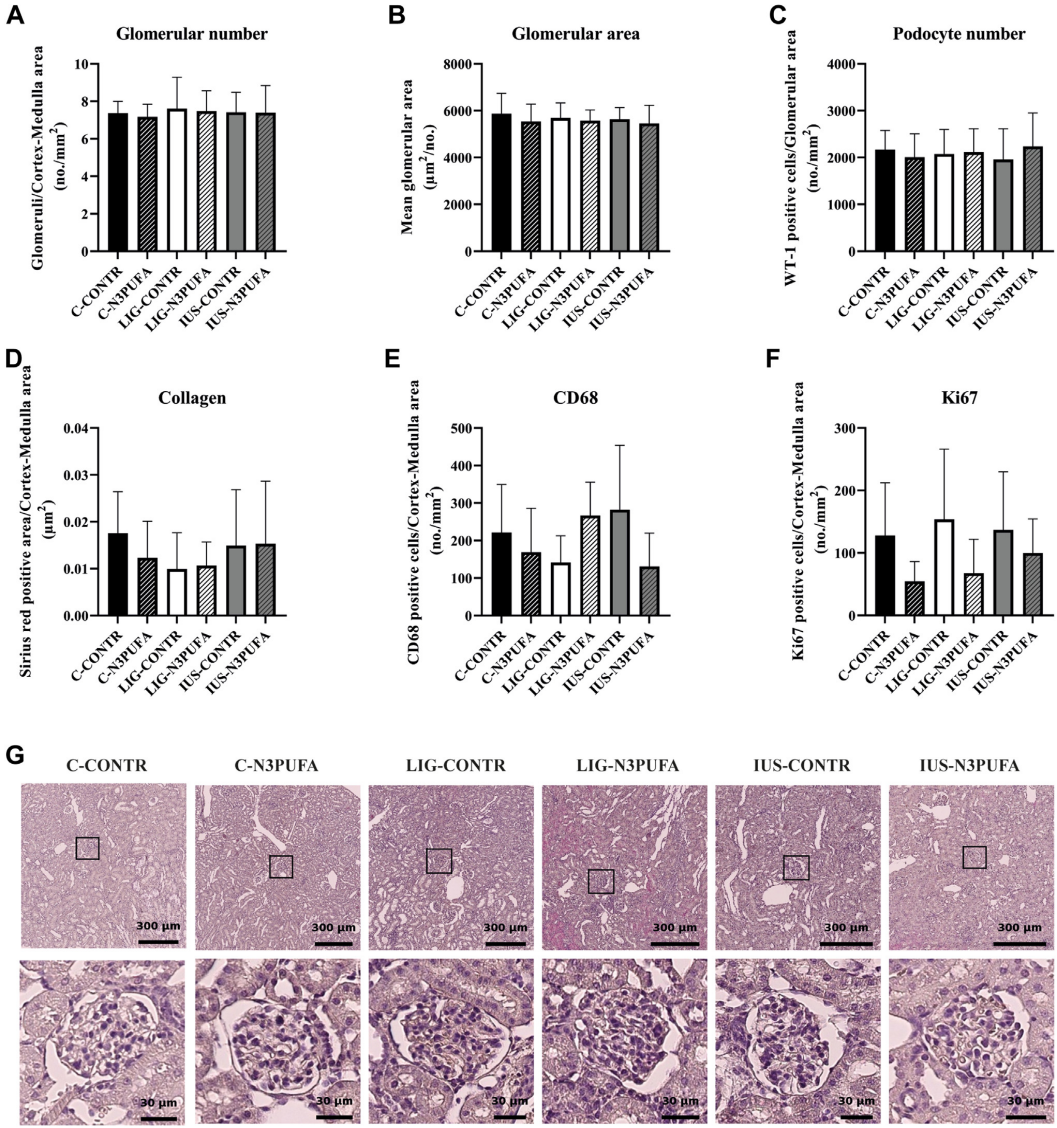
Assessment of standard kidney morphology in female rats on P39. A, B: Glomerular number and glomerular area were analyzed on H&E-stained slices. Representative H&E stains for each group are shown in (G). C: Podocyte number was assessed by WT-1 positive cells per glomerular area. D: Collagen deposition was analyzed using Sirius Red staining (positive staining per cortex-medulla area). E: CD68-positive cells per cortex-medulla area indicate the number of infiltrating monocytes and macrophages, and (F) Ki67-positive cells per cortex-medulla area indicate proliferating cells. Values are shown as mean ± SD, no significant differences (*P* < 0.05; Bonferroni-adjusted Mann-Whitney test) were observed in the presented analyses.

**Fig. 3:**
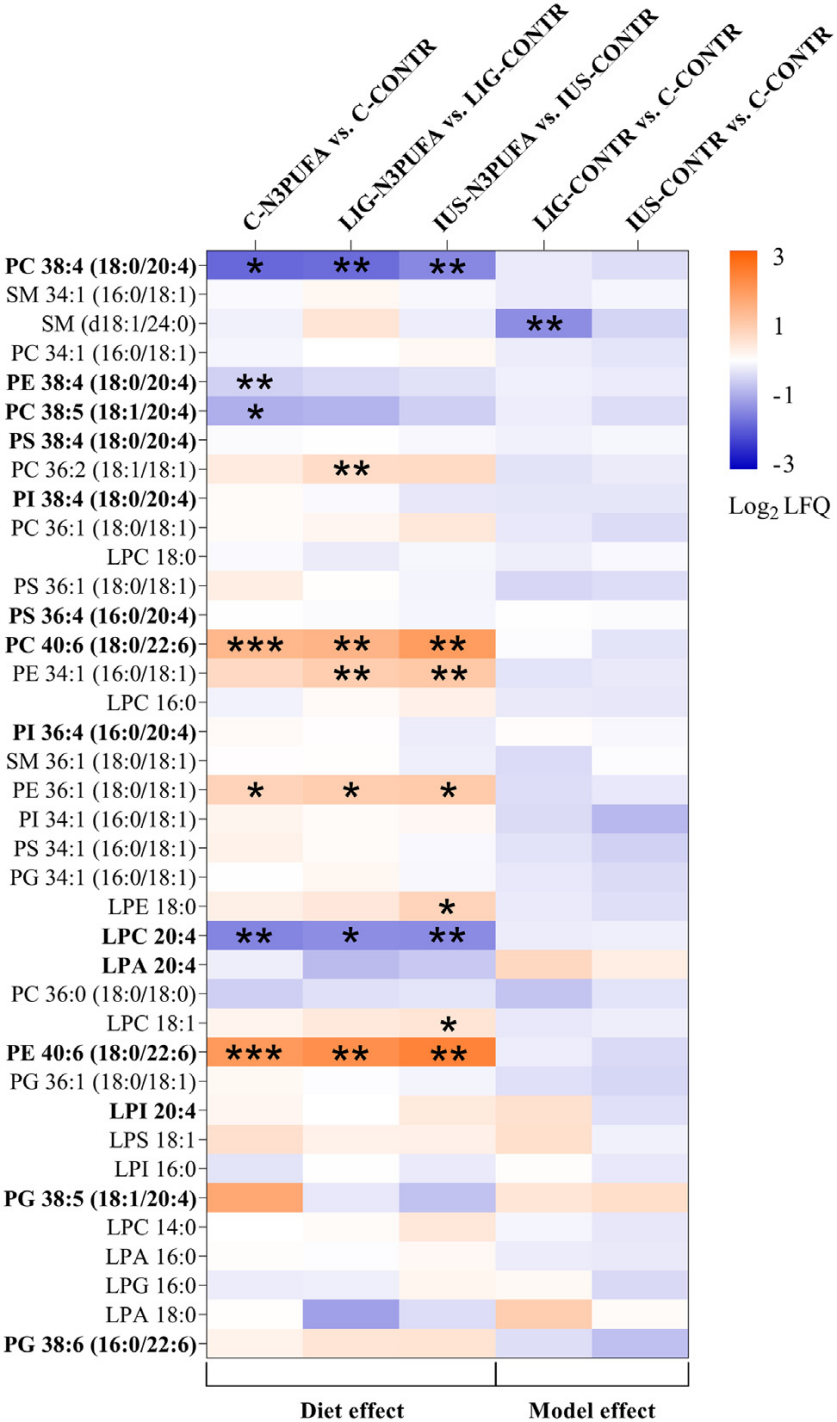
Heat map of PLs from female kidney cortex on P39. Parameters were sorted by their absolute concentrations in C-CONTR cortex tissue. Values are presented as Log2 label-free quantification of group differences (fc) for five different group comparisons (*n* = 6 rats per group). Absolute concentrations of PLs in all groups are shown in Table 3. Mann-Whitney test was performed for single group comparisons, and Bonferroni correction was applied. Adjusted *P* values of *P* < 0.05 were considered significant and marked with an asterisk (∗*P* < 0.05, ∗∗*P* < 0.01, and ∗∗∗*P* < 0.001).

**Fig. 4:**
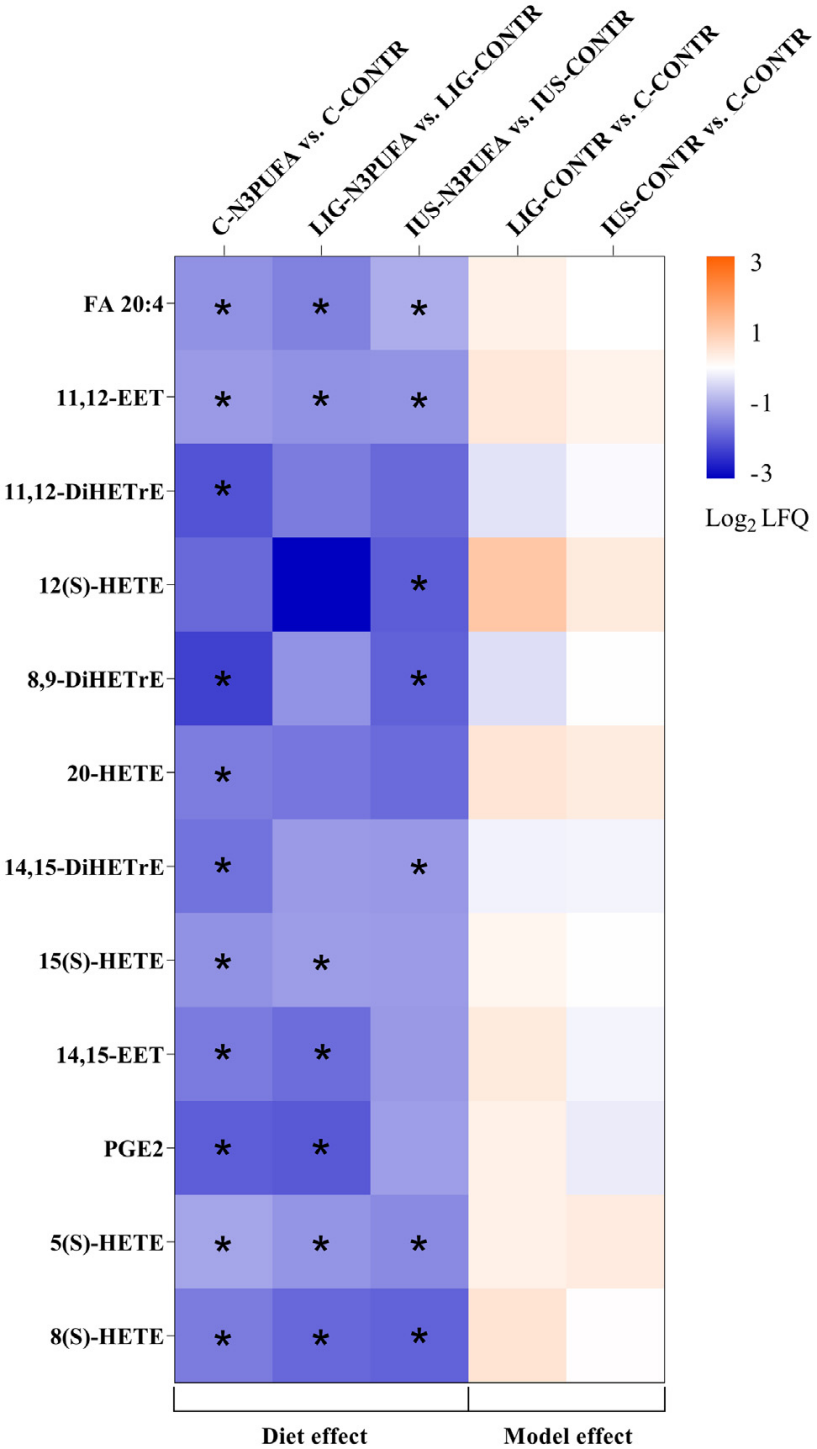
Heat map of AA metabolites from female kidney cortex on P39. Parameters were sorted by absolute concentrations in C-CONTR cortex tissue. Values are presented as Log2 label-free quantification of group differences (fc) for five different group comparisons (*n* = 6 rats per group). Absolute concentrations of AA metabolites in all groups are shown in Table 3. Mann-Whitney test was performed for single group comparisons, and Bonferroni correction was applied. Adjusted *P* values of *P* < 0.05 were considered significant and marked with an asterisk (∗*P* < 0.05).

### Kidney cortex proteomics

Snap-frozen kidney cortex (P39) was sectioned, and 5 mg was mixed with 150 μl of urea lysis buffer (8 M urea in 50 mM tetraethylammonium bromide). Chromatin was degraded, heated at 37°C for 30 min, and centrifuged. Samples were then incubated with 100 mM DTT at 25°C for 1 h. Afterward, 40 mM chloroacetamide was added and incubated for 30 min in the dark at room temperature. Lysyl endopeptidase at an enzyme/substrate ratio of 1:75 was added and incubated for 2 h at 25°C. The samples were diluted with 50 mM tetraethylammonium bromide to a final concentration of ≤2 M urea. Trypsin at an enzyme/substrate ratio of 1:75 was added and incubated overnight at 25°C. On the second day, 1% formic acid was added and continued with the StageTip purification of peptides. The StageTips were equilibrated with methanol and 0.1% formic acid in either water (buffer A) or 80% acetonitrile (buffer B). Samples were loaded onto the StageTips. The StageTips were washed once with 20 μl buffer A and twice with buffer B. In the end, StageTips were dried with a syringe and stored at −4°C before LC-MS analysis was performed by the staff of the CECAD Proteomics facility.

The MS system contains a Q Exactive Plus Orbitrap and an EASY nLC 1000 with a C18 analytical column (50–75 μm I.D., filled with 2.7 μm Poroshell EC120 C18). The detailed performance is available elsewhere (2.4, 2.5) (32).

Further analysis was performed by using the Perseus software (version 1.5.5.3) (33). Differences between groups in the cortex proteome of IUGR were analyzed as follows: *1*) LIG-CONTR versus C-CONTR, *2*) IUS-CONTR versus C-CONTR, and the comparison of the dietary impact within groups: *3*) C-N3PUFA versus C-CONTR, *4*) LIG-N3PUFA versus LIG-CONTR, and *5*) IUS-N3PUFA versus IUS-CONTR. Statistical analysis was performed by a one-way ANOVA and a two-sample *t*-test, and significance was reached at *P* < 0.05, without further correction for multiple testing. Significantly altered proteins with a log_2_ fold change (fc) of >|0.58| (fc > 1.5) were classified as relevantly altered and used for further data processing. The UniProt protein knowledgebase, the String Database (version 11.0) (34), FunRich (version 3.1.3) (35), and ShinyGO (version 0.75) (36) were used for data analysis and identification of involved pathways.

### Statistical analysis

For statistical analyses, GraphPad Prism software (version 9.0.2; GraphPad Software, Inc) was used. Unless otherwise mentioned, a nonparametric and unpaired Mann-Whitney test was performed with two groups. Thereafter, a Bonferroni correction was used for multiple comparisons, depending on the number of comparisons. In most cases, we adjusted for the following five comparisons (LIG-CONTR vs. C-CONTR, IUS-CONTR vs. C-CONTR, C-N3PUFA vs. C-CONTR, LIG-N3PUFA vs. LIG-CONTR, and IUS-N3PUFA vs. IUS-CONTR). An adjusted *P* value of <0.05 (≙unadjusted *P* < 0.01) was considered significant. Significant results are marked with asterisk (∗*P* < 0.05, ∗∗*P* < 0.01, and ∗∗∗*P* < 0.001). For details on statistical analyses of proteomics data, please refer to the respective paragraphs above.

## Results

### IUGR was induced by uteroplacental insufficiency and IUS, but postnatal catch-up growth was exclusively present after IUS

Birth weight (i.e., body weight on P2) was significantly reduced in female rat offspring both after uteroplacental insufficiency by LIG (*n* = 49, *P* < 0.001; LIG (<10th percentile), *n* = 39, *P* < 0.001) and IUS (*n* = 63, *P* = 0.039), indicating IUGR. Both groups were compared with unimpaired control offspring (C, *n* = 98; Fig. 1A). The 10th weight percentile of unimpaired C offspring was used as an additional cutoff in LIG offspring (23), corresponding to the human small for gestational age definition (24). To illustrate this in Fig. 1A, “LIG” includes all female LIG offspring independent of their body weight, whereas “LIG (<10th percentile)” represents female LIG offspring with a birth weight below the 10th weight percentile. Figure 1B shows birth weights of all female rat offspring included in the foster litters. Postnatal body weight gain of female LIG rats remained significantly lower compared with female C rats until the end point. Thus, LIG females showed no postnatal catch-up growth (body weight on P35, *P* = 0.010). However, in female IUS rats, significantly lower body weights were present at birth only (Fig. 1C).

### Triglyceride and total cholesterol plasma concentrations were reduced by early n-3 PUFA diet intervention primarily after IUS

Circulating triglycerides and total cholesterol were reduced in young adult female rats (P33) of all early diet intervention groups, but only the difference in triglycerides between the IUS-N3PUFA group and the IUS-CONTR group reached statistical significance (mean difference: −59.08 mg/dl, *P* < 0.001). In addition, IUS rats receiving diet intervention had significantly increased cystatin C (mean difference: 0.3 mg/l, *P* = 0.035) and decreased urea (−9.78 mg/l, *P* = 0.008) concentrations in plasma compared with controls receiving control diet. Other kidney and liver function-related parameters (total protein, creatinine, electrolytes, and alanine transaminase/aspartate transaminase) were not significantly different. For all comparisons of metabolic parameters in plasma, please see Table 2.

### Standard kidney morphology parameters (glomerular number, podocyte number per glomerulus, inflammatory cell number, and collagen deposition) were affected neither by IUGR nor by early n-3 PUFA diet intervention

To address the question whether IUGR or our early diet intervention have an impact on kidney morphology in early life, we assessed standard histological parameters on P39. Glomerular number (Fig. 2A) and area (Fig. 2B) were not significantly affected, neither by IUGR nor by the diet intervention. Similarly, cell numbers of podocytes per glomerulus (WT-1 staining; Fig. 2C) as well as kidney collagen deposition (Fig. 2D), monocytes and macrophages (CD68 staining; Fig. 2E), and proliferating cells (Ki67 staining; Fig. 2F) did not differ significantly between groups. Figure 3G shows representative H&E stains for each group. Thus, manifest kidney disease was excluded in all experimental groups. “Typical” nonreversible morphological alterations like a significantly reduced nephron number, which has been linked to an increased risk for CKD in IUGR individuals (37), were not present in our setting.

### Early n-3 PUFA diet intervention substantially downregulated PLs containing AA and strongly increased PLs containing DHA in kidney cortex tissue

Sustainable effects of modified dietary fatty acid composition are reflected in the PL composition of cell membranes (Fig. 3 and Table 3, upper half). Interestingly, neither IUGR nor early diet intervention altered the relative proportions of PL classes (data not shown).

As expected, AA (20:4) was the most abundant fatty acid in PLs of all groups. Interestingly, early n-3 PUFA diet intervention substantially reduced the absolute concentrations of AA in cell membranes. The single most regulated PL was PC 38:4 (PC containing 18:0 and 20:4n-6), which was decreased to 25% of the baseline concentration by diet in controls and in both IUGR models (C-N3PUFA vs. C-CONTR, *P* = 0.015; LIG-N3PUFA vs. LIG-CONTR, *P* = 0.003; and IUS-N3PUFA vs. IUS-CONTR, *P* = 0.004). A similar pattern was observed for lyso-phosphatidylcholine (LPC) 20:4n-6 (C-N3PUFA vs. C-CONTR, *P* = 0.002; LIG-N3PUFA vs. LIG-CONTR, *P* = 0.012; and IUS-N3PUFA vs. IUS-CONTR, *P* = 0.004). The amounts of PE38:4 (18:0, 20:4n-6) and PC 38:5 (18:1, 20:4n-6) were also downregulated in C-N3PUFA versus C-CONTR (*P* = 0.009, *P* = 0.036).

In contrast, PL with DHA (22:6n-3), such as PC 40:6 (18:0, 22:6n-3), was elevated 2- to 3-fold in all three groups via intervention diet (C-N3PUFA vs. C-CONTR, *P* < 0.001; LIG-N3PUFA vs. LIG-CONTR, *P* = 0.003; and IUS-N3PUFA vs. IUS-CONTR, *P* = 0.004). This effect was also seen in PE 40:6 (18:0, 22:6n-3; C-N3PUFA vs. C-CONTR, *P* < 0.001; LIG-N3PUFA vs. LIG-CONTR, *P* = 0.003; and IUS-N3PUFA vs. IUS-CONTR, *P* = 0.004).

Furthermore, concentrations of PLs containing stearic acid (18:0) and oleic acid (18:1) were predominantly regulated in IUGR groups. Thus, concentrations of PC 36:2 (18:1, 18:1), phosphatidylethanolamine (PE) 34:1 (16:0, 18:1), and PE 36:1 (18:0, 18:1) were upregulated in LIG-N3PUFA (*P* = 0.009, *P* = 0.009, and *P* = 0.011) and concentrations of PE 34:1 (16:0, 18:1), lyso-phosphatidylethanolamine 18:0, LPC 18:1, and PE 36:1 (18:0, 18:1) were elevated in IUS-N3PUFA rats (*P* = 0.009, *P* = 0.036, *P* = 0.018, and *P* = 0.011). Only PE 36:1 (18:0, 18:1) was also elevated in C-N3PUFA versus C-CONTR rats.

Interestingly, SM was decreased after LIG only (d18:1, 24:0; *P* = 0.006) and not affected by early diet (Fig. 3).

### Early n-3 PUFA diet intervention resulted in a strong reduction in AA-derived mediators in kidney cortex tissue

Beyond PL composition, we also studied AA-derived mediators in kidney cortex tissue to analyze the impact of early n-3 PUFA diet intervention on lipid-derived effector molecules (Fig. 4 and Table 3, lower half). Diet intervention resulted in a strong downregulation of the total amount of tissue AA (20:4) in both the controls and IUGR groups (C-N3PUFA vs. C CONTR, *P* = 0.0108; LIG-N3PUFA vs. LIG-CONTR, *P* = 0.0108; and IUS-N3PUFA vs. IUS CONTR, *P* = 0.0216). In addition, our diet intervention led to a decrease in eicosanoid metabolites 5(S-), 8(S)-, 12(S)-, 15(S)-, and 20-HETE. In controls, the early n-3 PUFA diet also downregulated DiHETrE (3×, *P* = 0.0108), whereas no differences of DiHETrEs were observed in IUGR rats. In LIG, our diet intervention decreased the amount of 11,12-, and 14,15-EET (*P* = 0.0216, *P* = 0.0216). PGE_2_ levels were reduced by our diet intervention in both C (*P* = 0.0433) and LIG (*P* = 0.0433) rats. IUGR without early diet intervention had no significant effect on AA metabolites (Fig. 4, two columns on the right show the “IUGR model effect”).

### Early n-3 PUFA diet intervention effectively reversed an adverse proteomic signature indicating inflammation and hypercoagulability in kidney cortex tissue after uteroplacental insufficiency by LIG

**Fig. 5:**
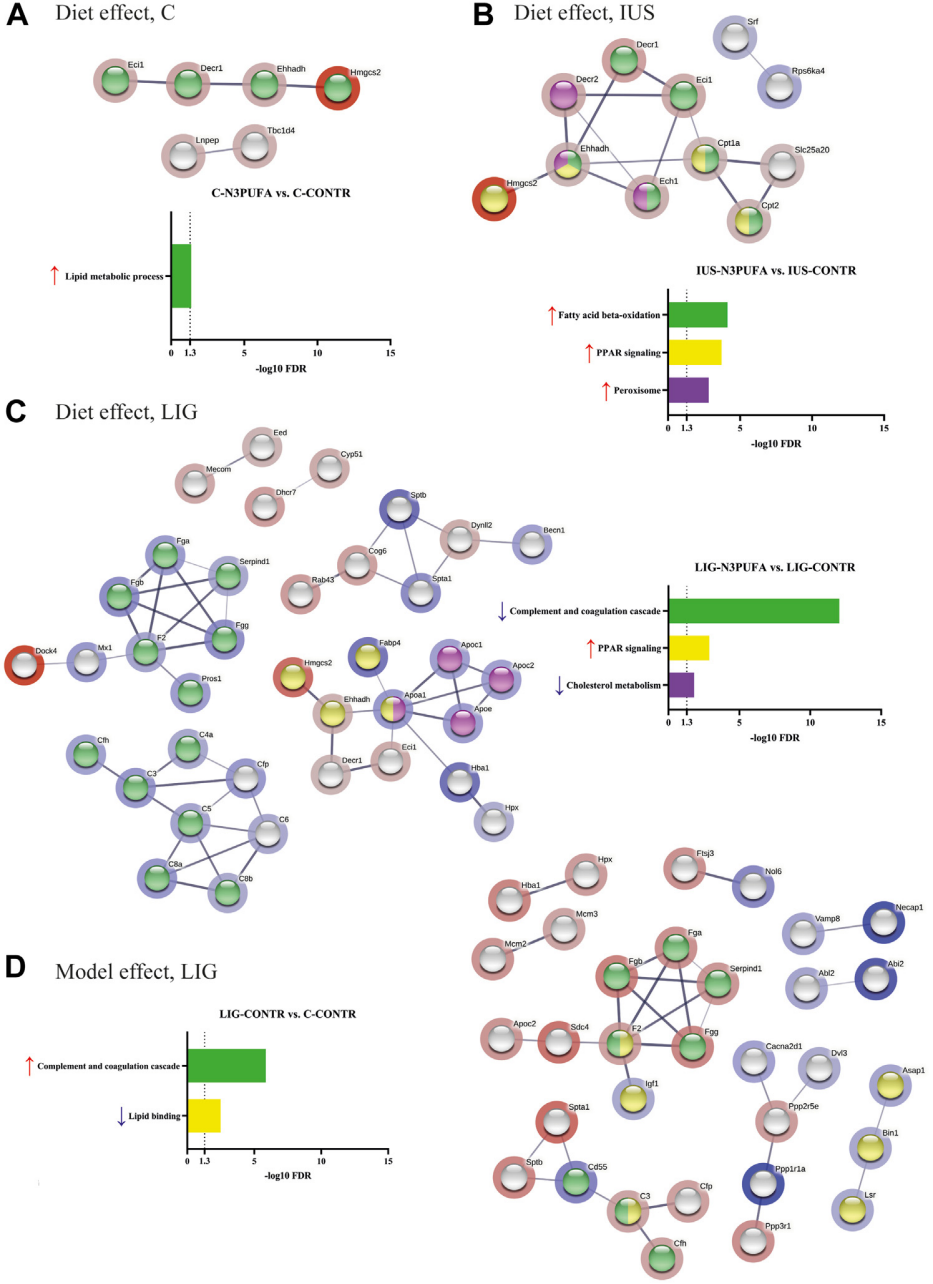
String analysis of the female kidney cortex proteome on P39. A: C-N3PUFA versus C-CONTR, (B) IUS-N3PUFA versus IUS-CONTR, (C) LIG-N3PUFA versus LIG-CONTR, and (D) LIG-CONTR versus C-CONTR. Group comparison IUS-CONTR versus C-CONTR is shown in supplemental Fig. 1. Colored nodes represent Kyoto Encyclopedia of Genes and Genomes pathways or GO terms (Lipid metabolic process, GO: 0006629; fatty acid beta-oxidation, GO: 0006635; PPAR signaling, rno03320; peroxisome, rno04146; complement and coagulation cascade, rno04610; cholesterol metabolism, rno04979; lipid binding, GO: 0008289) with a –log10 false discovery rate of >1.3. Intensity of background shadings is related to the fc of each protein. Red shading indicates increased protein expression, and blue shading indicates decreased protein expression within an fc >1.5.

Shotgun proteomics were performed from kidney cortex tissue to analyze the “model effect” (i.e., the comparisons LIG-CONTR vs. C-CONTR and IUS-CONTR vs. C-CONTR) and the “diet effect” (i.e., the comparisons C-N3PUFA vs. C-CONTR, LIG-N3PUFA vs. LIG-CONTR, and IUS-N3PUFA vs. IUS-CONTR) on the renal proteome. First, “relevantly” altered proteins were defined as significantly altered proteins (*P* < 0.05) with a log_2_ fc of >|0.58| (i.e., fc > 1.5). In the following analyses, only proteins that met these criteria were included. In Supplemental Table 3, the top 10 upregulated and downregulated proteins for all group comparisons are listed. Figure 5 shows functional String analyses of proteins relevantly altered divided into “diet effect” and “IUGR model effect.” Comparison of IUS-CONTR versus C-CONTR yielded a very small network only, which is presented in supplemental Fig. 1.

Analysis of the “diet effect” revealed a cluster of proteins shared by all diet intervention groups regardless of IUGR, which was related to the Gene Ontology (GO) terms *lipid metabolic process*, *fatty acid ß-oxidation*, *PPAR signaling*, and *peroxisome*. In detail, the expression of Ehhadh, Hmgc2, Decr1/2, and Eci1 was increased in all offspring groups. Hmgc2 is a rate-limiting enzyme for ketogenesis (38) and was the top upregulated protein in this cluster. Upregulations of Ehhadh, DECR1/2, and Eci1 increase β-oxidation of unsaturated fatty acids in mitochondria and peroxisomes (39). Interestingly, a larger number of diet-affected proteins were assignable to clusters in the IUGR groups than in the control group (LIG >> IUS > C). In IUS rats, in addition to the common cluster described above, the mitochondrial transporters for fatty acids Cpt1a, Cpt2, and Slc25a20 were upregulated and coclustered with the “common” cluster. In rats after uteroplacental insufficiency by LIG, the number of protein clusters was particularly high. In these rats, the “common” cluster coclustered with Apoa1, Apoc1, Apoc2, Apoe, and Fabp4, which were all downregulated and related to the *cholesterol metabolism* or *PPAR signaling*. A second cluster included Fga, Fgb, Fgg, F2, Pros1, and Serpind1. A third cluster included C3, C4a, C5, C6, C8a, C8b, Cfp, and Cfh. All proteins from clusters 2 and 3 showing the diet effect in LIG were downregulated and related to the *complement and coagulation cascade*. Three other small clusters were also identified but could not be assigned to significant GO terms.

Analysis of the “IUGR model effect” in LIG (LIG-CONTR vs. C-CONTR) yielded a first cluster including Arf-GTPase-activating protein 1 (Asap1), Bin1, and lipolysis-stimulated lipoprotein receptor (Lsr), which were all downregulated and related to *lipid binding*. In addition, a second cluster including Fga, Fgb, Fgg, F2, Serpind1, Igf1, Sdc4, and Apoc2 as well as a third cluster including C3, Cfp, Cfh, Cd55, Sptb, and Spta1 could be identified. Clusters 2 and 3 showing the LIG effect were again related to the Kyoto Encyclopedia of Genes and Genomes pathway *complement and coagulation cascade* and the GO term *lipid binding*.

**Fig. 6:**
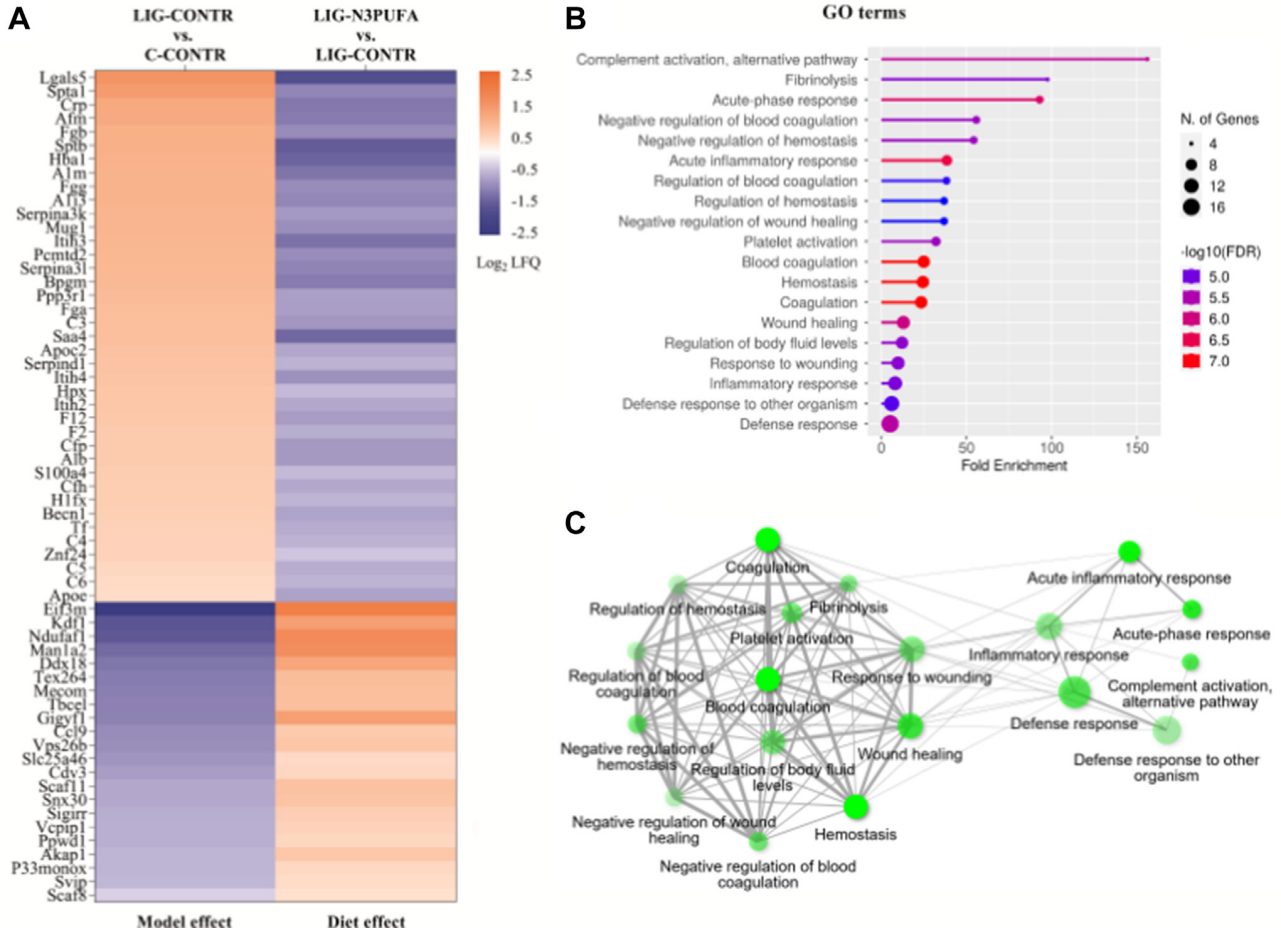
Kidney cortex proteins that were differentially expressed in LIG (LIG-CONTR vs. C-CONTR, *P* < 0.05) on P39 and reversely regulated by early n-3 PUFA diet in LIG, (LIG-N3PUFA vs. LIG-CONTR, *P* < 0.05). A: Heat map of 61 proteins differentially expressed in LIG (LIG-CONTR vs. C-CONTR) and reversely altered in LIG by the diet intervention (LIG-N3PUFA vs. LIG-CONTR). B: Analysis of the reversely altered proteins with ShinyGo; lollipop chart of the top 20 related GO terms (biological process) sorted by false discovery rate (FDR). Size of the nodes represents the number of regulated proteins (larger nodes indicate a higher number; also see legend in the figure), color represents FDR (also see legend in the figure), length of lollipop represents fold enrichment. C: Network analysis of the GO terms identified by ShinyGo. Larger nodes again represent a higher number of regulated proteins, high color intensity represents small FDR, and thicker lines represent more overlapping proteins.

Of note, 61 proteins that were identified to be significantly dysregulated by one of our IUGR models (LIG-CONTR vs. C-CONTR) were reversely altered by the diet intervention (LIG-N3PUFA vs. LIG-CONTR) (Fig. 6A). Further analysis of these proteins by ShinyGo analysis (Fig. 6B) identified two big GO term clusters (Fig. 6C). One cluster identified inflammation-related pathways like *Complement activation*, *response to cytokine* or *innate immune response*. The other one clustered coagulation-related pathways like *blood coagulation*, *fibrinolysis*, or *platelet activation*.

## Discussion

Our study provides first evidence that the potential efficacy of a larger n-3/n-6 PUFA ratio in early life diet in the prevention of kidney disease depends on whether an individual had been exposed to normal or adverse environmental conditions prenatally. A proinflammatory and hypercoagulable protein signature after IUGR because of uteroplacental insufficiency can be reversed by an n-3/n-6 ratio of 1:1 in young adult rats. In addition, N3PUFA diet intervention strongly reduces proinflammatory PL-AA and AA metabolites while increasing anti-inflammatory PL-DHA in kidney cortex tissue regardless of intrauterine conditions.

The IUGR rat models of LIG and IUS are well established. Our group (22, 40) and others (41, 42) have shown that both models impair intrauterine weight gain, modify nephrogenesis, and increase the risk for impaired kidney function in later life (12, 41, 42, 43, 44). However, metabolic programming effects also depend on the exact cause of IUGR and may differ depending on postnatal conditions (40). Therefore, we first analyzed the effects of our two IUGR models (“IUGR effect”) and of the diet intervention (“diet effect”) on standard blood parameters and standard morphological analyses of the kidney during and at the end of the diet intervention period (P33, P39). Basically, IUGR had no major effect on these parameters. Pronounced morphological or metabolic alterations had not been expected, since prior studies showing kidney damage in the respective IUGR models had analyzed kidneys later in life or after a second hit (12, 21). In addition, we did not reduce litter sizes to less than eight pups in this study in order to prevent postnatal overfeeding. Thus, in contrast to prior studies, we did not observe catch-up growth, which is a major risk factor for metabolic sequelae “on top” of being born IUGR (45). Nevertheless, a small effect of the diet intervention on plasma triglyceride concentrations could be demonstrated, which were lower in all groups following diet intervention although only the comparison of IUS-N3PUFA and IUS-CONTR reached statistical significance. In the literature, decreased lipogenesis in the liver is discussed as the most probable cause for triglyceride-lowering effects of omega-3 enriched diets (46). Our observation is also consistent with clinical effects of n-3 fatty acid substitution. In children and adolescents with obesity, n-3 fatty acids led to a reduction of triglycerides by 39% (47). Together, these data highlight the potential of n-3 PUFAs to modify circulating triglyceride concentrations in early life.

We then looked at tissue lipid composition. In prior studies, an inter-relationship of dietary fatty acid supply and the amount of single fatty acids in kidney cell membranes had been described in 3-month-old rats (48) and as early as during lactation in suckling offspring (18). The additional value of our study is the separate analysis of individuals with (i.e., IUGR) and without (i.e., C) susceptibility toward kidney disease. Our intervention diet was fed from P2 to 39 to fully cover postnatal kidney development, maturation, and growth in rats (49, 50, 51). Neither of our diets contained much AA. Nevertheless, AA was the most abundant fatty acid in kidney cortex PLs as expected (52). Thus, our data confirm that AA is predominantly derived from enzymatic conversion (53). Since AA is mainly synthesized from linoleic acid (LA, 18:2) (53), the 35% reduction of LA content in our n-3 PUFA diet will have contributed to the reduction of PLs containing AA in kidney cortex tissue of the diet intervention groups. In contrast to the high conversion rate of LA to AA, the conversion ratio of alpha-linolenic acid (18:3) into EPA and DHA is marginal, in some studies less than 1% (54). Thus, alpha-linolenic acid-enriched diet does not significantly increase PLs containing EPA and DHA within rat kidney tissue (48). Therefore, absolute dietary concentrations of EPA and DHA are essential information beyond n-3/n-6 ratios when analyzing n-3 PUFA effects. The dietary supply of choline did not influence the total amount of investigated choline-derived PLs in the kidney. However, choline was mainly added to the diet to increase bioavailability of DHA, since choline has a major impact on the transport of fatty acids through the periphery. Studies have shown synergistic effects of choline and DHA in the accumulation of DHA in cell membranes (55).

Looking at diet effects in detail, the major PLs containing AA (i.e., PC 38:4, LPC 20:4) were strongly downregulated by the N3PUFA diet. On the contrary, major PLs containing DHA (i.e., PC 40:6, PE 40:6) were markedly elevated in all N3PUFA diet intervention groups. Importantly, certain PLs containing stearic acid (18:0) and oleic acid (18:1) were more strongly influenced in IUGR than in control groups by early life diet. These changes are highly relevant in a pathophysiological context. Fatty acid composition of PLs influences cell membrane integrity and properties (56, 57). Furthermore, AA metabolites are a driving force of inflammation (58), endothelial dysfunction, and oxidative stress (59). Membrane AA is metabolized to lipid mediators via three major classes of enzymes. First, cyclooxygenase enzymes may convert AA into prostanoids, which mediate inflammation and impact upon vascular function, blood pressure homeostasis, and platelet aggregation (60). PGE_2_ levels were significantly reduced by N3PUFA diet in C and LIG offspring. Under physiological conditions, PGE_2_ is the most abundant prostanoid in cortex and medulla tissue. It acts as a vasodilator in renal vessels and thereby stabilizes renal blood flow and glomerular filtration rate (61). In addition, PGE_2_ is a proinflammatory mediator highly inducible by PGE_2_ synthase (62). Therefore, it is also a potent mediator in podocyte and tubular disease and contributes to renal injury (63, 64, 65, 66). In CKD, PGE_2_ induction contributes to morphological changes of podocytes, making the filtration barrier more permeable (66). Second, cytochrome P450 enzymes (CYP enzymes) can convert AA into EETs and HETEs. EETs are rapidly metabolized to DiHETrE via soluble epoxide hydrolases (57). Kidneys produce relative high amounts of EETs, which are known to have vasodilator and anti-inflammatory effects similar to DHA-derived metabolites (67). In the renal cortex, the predominant EETs are 14,15-EET and 11,12-EET (68). In our study, both 11,12-EET (C, LIG, IUS) and 14,15 EET (C, LIG), as well as 8,9-DiHETrE (C, IUS), 11,12-DiHETrE (C), and 14,15-DiHETrE (C, IUS) were downregulated following n-3 PUFA diet in early life. Since DiHETrEs were predominantly downregulated in C rats, we speculate that soluble epoxide hydrolase activity might differ between C and IUGR rats (69). Third, lipoxygenase enzymes convert AA into HETE and leukotriene A_4_. In our study, 12(S)-HETE was downregulated in IUS and 15(S)-HETE was downregulated in C and LIG rats following n-3 PUFA diet. In rat glomeruli, 12-HETE is the main HETE subtype being produced. 12-HETE may act as a proinflammatory mediator (70). Via the expression of eNOS, 12-HETE and 15-HETE are important regulators of blood pressure, and downregulation of these mediators is effective in alleviating hypertension (30, 71). As stated above, PLs containing DHA were elevated after N3PUFA diet. DHA metabolites act antagonistically to AA and AA metabolites (18, 72, 73). Via lipoxygenase enzymes, DHA is metabolized to D-series resolvins, protectins, and maresins, which are involved in actively limiting inflammation (74). In addition, epoxydocosapentaenoic acids and dihydroxydocosapentaenoic acids are produced via CYP pathways and are potent in lowering blood pressure (73). In experimental acute kidney injury, specialized proresolving mediators derived from DHA had a protective effect on the severity of kidney injury going along with a reduction in fibrosis, infiltration of leukocytes, and activation of macrophages (75). Unfortunately, despite increased concentrations of DHA-containing PLs in our study, DHA-derived effector molecules were below the detection limit. Interestingly, fatty acids from cell membranes are released by different subtypes of phospholipase A_2_ (PLA_2_). Studies suggest that DHA-enriched cell membranes may inhibit cytosolic PLA_2_ to release AA (76, 77). In mesangial cells, administration of n-3 fatty acid derivatives (AVX001/2) led to a reduction of cPLA_2_- and PGE_2_-activity via interleukin-1β (12). Thus, lower PLA_2_ activity might add to our finding of smaller amounts of AA-derived eicosanoids following N3PUFA diet in addition to reduced absolute concentrations of PLs containing AA. In a further step, we analyzed whether there is an independent IUGR effect on tissue lipids. Only SM (d18:1/24:0) was strongly and significantly downregulated in LIG compared with C rats. SMs are part of lipid rafts involved in lipid mediator signaling via the binding of proteins to the membrane (49). No other PLs in kidney tissue were regulated by IUGR. In an earlier study, PC 36:2, LPC 18:1, and PC 18:2 were shown to be reduced in the plasma of LIG rats compared with controls (20). Unfortunately, we did not analyze plasma lipidomics in our study, since we were interested in kidney-specific effects. However, PC 36:2 was upregulated by n-3 PUFA diet in kidney tissue of LIG. Together, these data demonstrate that PC 36:2 is a PL in kidney tissue susceptible to regulations by early life conditions.

We then went on to find out whether we can also demonstrate a diet effect on the proteome level. For that purpose, we first analyzed the IUGR effect on the proteome. Two-thirds of the regulated proteins in LIG versus C compared with IUS versus C were group specific. No shared functional clusters were observed. In prior studies, we had already demonstrated that molecular signatures during and at the end of nephrogenesis differ between LIG and IUS offspring (20, 40). In the current study, differentially expressed proteins were mainly found in LIG and clustered within the Kyoto Encyclopedia of Genes and Genomes pathways *Complement and coagulation cascade* and the GO term *lipid binding*. This is in line with a prior study in which IUGR was induced by low protein. In neonatal low protein kidneys, several complement-associated genes and coagulation-linked *serpin* genes were elevated (78). An enhanced coagulation cascade correlates with impaired kidney function, especially in CKD (79). Thus, the protein signature found in LIG may contribute to impaired microcirculation and vascular dysregulation via higher blood viscosity, enhanced endothelial reactivity, and impaired endothelial integrity (80). More specifically, the proteomic signature found in LIG might predispose to the development of thrombotic microangiopathy (TMA). TMA is a situation in which microvascular thrombosis results from complement activation and other causes of endothelial injury such as hypertension or antibody-mediated allograft rejection. TMA aggravates the course of kidney diseases like nephrotic syndrome (81) or IgA nephropathy (82). Interestingly, LIG rats develop elevated blood pressure and a dysregulated vascular tone in interlobar arteries on P70 (83). In addition, they are at risk for a more severe progression of IgA nephritis (21). Within the GO term *lipid binding*, Lsr was downregulated in LIG. Lsr may decrease the elimination of lipoprotein particles rich in triglycerides (84). In addition, Asap1 was downregulated, which hints at less phosphatidylinositol 4,5-bisphosphate activation. Reduced phosphatidylinositol 4,5-bisphosphate activation in turn could lead to a reduction in PL binding activity and membrane remodeling (85). Finally, a reduced expression of Igf1 (in LIG, but also in IUS) could be related to an increased risk for insulin resistance and cardiovascular diseases (86).

Next, we analyzed the diet effect on the proteome. Our data demonstrate a general IUGR-independent effect of the diet intervention on key proteins in lipid metabolism related to the GO terms *lipid metabolic process*, *fatty acid ß-oxidation*, *PPAR signaling*, and *peroxisome*. Consequently, a diet enriched in n-3 PUFAs and choline may enhance β-oxidation, excess the accumulation of acetyl-CoA, and enhance the synthesis of β-hydroxybutyrate (38, 39). This finally might increase the proportion of ketone bodies for energy supply in kidneys (87). Ketone bodies may have beneficial effects during oxidative stress, inflammation, cell death, and interstitial fibrosis (88), that is, in diabetic kidney disease (89). Excitingly, beyond general effects of the diet intervention, we were able to demonstrate an IUGR-specific effect of n-3 PUFA diet on proteomic signatures in LIG rats. More specifically, N3PUFA diet had an opposite effect on 61 of 170 differentially expressed proteins in LIG. Most of these proteins were related to the GO terms *blood coagulation*, *acute inflammatory response*, *complement activation*, or *response to cytokines*. Furthermore, prominent upregulation of C-reactive protein and protein S100-A4 (S100a4) indicative of a proinflammatory signature in LIG was abolished by the diet intervention.

As a consequence, n-3 PUFA diet may decrease the risk for impaired microcirculation, TMA, and vascular dysregulation in LIG offspring. A therapeutic potential of n-3 PUFA to inhibit the coagulation cascade and lower the risk for cardiovascular disease has been discussed before (90). However, we are the first to demonstrate that a time-limited dietary intervention during early life might be sufficient to reverse adverse proteomic signatures.

Our study has some limitations. First, we only included female offspring, since male offspring were used for separate research questions later in life. Second, we did not perform Bonferroni adjustment of the proteome analysis, which would have accounted for multiple comparisons. However, in the context of perinatal programming, differences in protein expression are rather small. Clinically relevant signatures may be eliminated by Bonferroni adjustment although small differences in early life may end up in CKD (6, 7). Furthermore, we only used kidney cortex tissue for omics analyses and can therefore not account for cell-specific alterations. Finally, it will have to be established by future studies whether the diet effects on protein and lipid signatures will indeed protect against kidney damage in later life.

In conclusion, our study provides evidence that an elevated n-3/n-6 PUFA ratio in early diet strongly reduces proinflammatory PLs and mediators while increasing DHA-containing PLs regardless of prior intrauterine conditions. Counteracting a proinflammatory hypercoagulable protein signature in young adult IUGR individuals through early diet intervention may be a feasible strategy to prevent developmentally programmed kidney damage in later life. Furthermore, our data identified a “window of opportunity” following adverse intrauterine kidney programming for preventive nutritional strategies, prior to loss of kidney function. It highlights the potential for personalized nutrition when treating patients at risk.

## Data availability

All data are contained within the article.

## Supplemental data

This article contains supplemental data.

## Conflict of interest

The authors declare that they have no conflicts of interest with the contents of this article.
