# Supplementary material for: Elevated n-3/n-6 PUFA ratio in early life diet reverses adverse intrauterine kidney programming in female rats
Source: J Lipid Res. 2022 Sep 21;63(11):100283. doi: 10.1016/j.jlr.2022.100283 (PMC9619183; doi:10.1016/j.jlr.2022.100283)
Supplement: Supplemental Tables S1–S3 and Figure S1 [file mmc1.docx]

**SUPPLEMENTAL INFORMATION:**

**Elevated n-3/n-6 PUFA ratio in early life diet reverses adverse intrauterine kidney programming in female rats.**

Jenny Voggel^1,2^, Gregor Fink^1^, Magdalena Zelck^1^, Maria Wohlfarth^1^, Julia M. Post^3^, Laura Bindila^3^, Manfred Rauh^4^, Kerstin Amann^5^, Miguel A. Alejandre Alcázar^1,2,6,7^, Jörg Dötsch^1^, Kai‑Dietrich Nüsken^1^, Eva Nüsken^1^

^1^Clinic and Polyclinic for Paediatric and Adolescent Medicine, University of Cologne, Faculty of Medicine and University Hospital Cologne, Germany; ^2^Center for Molecular Medicine Cologne (CMMC), University of Cologne, Cologne, Germany; ^3^Institute of Physiological Chemistry, University Medical Center of the Johannes Gutenberg University of Mainz, Mainz, Germany; ^4^Department of Pediatrics and Adolescent Medicine, University Hospital Erlangen, Erlangen, Germany; ^5^Department of Nephropathology, Institute of Pathology, Friedrich-Alexander-University Erlangen, Erlangen, Germany; ^6^Cologne Excellence Cluster on Cellular Stress Responses in Aging-Associated Diseases (CECAD), University of Cologne, Cologne, Germany; ^7^Institute for Lung Health, University of Giessen and Marburg Lung Center (UGMLC), Member of the German Center for Lung Research (DZL), Gießen, Germany.

**Supplemental Table 1:** Assay information from measured plasma parameters.

| Parameter | Assay type | Roche Cobas® Reagents | CV% | LOD |
| --- | --- | --- | --- | --- |
| Albumin | Colometry (Bromocresol green) | ALB2 | 3.0 | 2.00 g/L |
| ALT | Photometry (IFCC) | ALTLP | 2.4 | 5.00 U/L |
| AST | Photometry (IFCC) | ASTLP | 2.8 | 5.00 U/L |
| Calcium | Photometry (NM-BAPTA) | CA2 | 1.8 | 0.20 mmol/L |
| Cholesterol | Enzymatic colorimetric assay | CHOL2 | 3.6 | 3.86 mg/dL |
| Creatinine | Enzymatic colorimetric assay | CREP2 | 1.5 | 0.06 mg/dL |
| Cystatin | Partice-enhanced turbidimetric immunoassay | CYSC2 | 1.6 | 0.40 mg/L |
| HDL | Homogeneous enzymatic colorimetric assay | HDLC4 | 2.3 | 3.09 mg/dL |
| Phosphate | Photometry | PHOS2 | 2.2 | 0.10 mmol/L |
| Potassium | Indirect ion selective electrode method | ISE indirect NA-K-CL for Gen.2 | 2.0 | - |
| Sodium | Indirect ion selective electrode method | ISE indirect NA-K-CL for Gen.2 | 1.6 | - |
| Total protein | Turbidimetry | TP2 | 1.9 | 2.00 g/L |
| Triglycerides | Enzymatic colorimetric assay | TRIGL | 1.7 | 8.85 mg/dL |
| Urea | Kinetic assay (urease/glutamate dehydrogenase) | UREAL | 1.4 | 30.00 mg/L |

CV%, coefficient of variation; LOD, limit of detection.

**Supplemental Table 2:** Internal Standards for lipidomic analysis.

| Lipid Standard | Internal Standard | Final concentration ISTDs in the lipid extract (ng/mL) |
| --- | --- | --- |
| LPA 18:0 | LPA 17:0 | 100 |
| LPA 20:4 |  |  |
| LPC 14:0 | LPC 17:0 | 100 |
| LPC 16:0 |  |  |
| LPC 18:0 |  |  |
| LPC 18:1 |  |  |
| LPC 20:4 |  |  |
| LPE 18:0 | PE 17:0-14:1 | 150 |
| LPG 16:0 | PG 17:0-14:1 | 100 |
| LPI 16:0 | PI 17:0-14:1 | 100 |
| LPI 20:4 |  |  |
| LPS 18:1 | PS 17:0-14:1 | 100 |
| PC 34:1 | PC 17:0-14:1 | 150 |
| PC 36:0 |  |  |
| PC 36:1 |  |  |
| PC 36:2 |  |  |
| PC 38:4 |  |  |
| PC 38:5 |  |  |
| PC 40:6 |  |  |
| PE 34:1 | PE 17:0-14:1 | 150 |
| PE 36:1 |  |  |
| PE 38:4 |  |  |
| PE 40:6 |  |  |
| PG 34:1 | PG 17:0-14:1 | 100 |
| PG 36:1 |  |  |
| PG 38:5 |  |  |
| PG 38:6 |  |  |
| PI 34:1 | PI 17:0-14:1 | 100 |
| PI 36:4 |  |  |
| PI 38:4 |  |  |
| PS 34:1 | PS 17:0-14:1 | 100 |
| PS 36:1 |  |  |
| PS 36:4 |  |  |
| PS 38:4 |  |  |
| SM 34:1 | SM d18:1/12:0 | 100 |
| SM 36:1 |  |  |
| 11,12-EET | 11,12-DiHETrE-d11 | 4 |
| 14,15-EET | 11,12-DiHETrE-d11  5(S)-HETE-d8 | 4  4 |
| 8,9-DiHETrE |  |  |
| 11,12-DiHETrE |  |  |
| 14,15-DiHETrE |  |  |
| 5(S)-HETE |  |  |
| 8(S)-HETE | 12(S)-HETE-d4 | 4 |
| 12(S)-HETE | 12(S)-HETE-d4  20-HETE-d6 | 4  4 |
| 15(S)-HETE |  |  |
| 20-HETE |  |  |
| PGE2 | PGE2-d4 | 2 |

DiHETrE, dihydroxy-eicosatrienoic acid; EET, epoxyeicosatrienoic acid; HETE, hydroxyeicosatetraenoic acid; LPA, lyso-phosphatidic acid; LPC, lyso-phosphatidylcholine; LPE, lyso-phosphatidylethanolamine; LPG, lyso-phosphatidylglycerol; LPI, lyso-phosphatidylinositol; LPS, lyso-phodphatidylserine; PC, phosphatidylcholine; PE, phosphatidylethanolamine; PG, phosphatidylglycerol; PGE2, prostaglandin E2; PI, phosphatidylinositol; PL, phospholipid; PS, phosphatidylserine; SM, sphingomyelin.

**Supplemental Table 3:** The 10 most up- and downregulated kidney cortex proteins on P39.

| Significantly altered kidney cortex proteins on P39 | | | Statistics | | 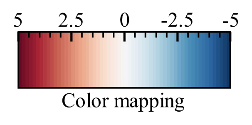 Heat map, log_2_ fc | | | | |
| --- | --- | --- | --- | --- | --- | --- | --- | --- | --- |
| Protein | *Gene* | Protein description | p-value | Log_2_ fc | Model effect | | Diet effect | | |
|  |  |  |  |  | LIG  § | IUS  $ | C  # | LIG  Δ | IUS  † |
| Q496Z1; Q712J3 | *Bicd2* | BICD cargo adaptor 2 | 0.0140^§^  0.0116^#^ | 2.30^§^  2.37^#^ | * |  | * |  |  |
| GOGA7 | *Golga7* | Golgin subfamily A member 7 | 0.0217^§^  0.0213^#^ | 1.82^§^  1.83^#^ | * |  | * |  |  |
| LEG5 | *Lgals5* | Galectin | 0.0476^§^  0.0032^Δ^ | 1.60^§^  -1.88^Δ^ | * |  |  | ** |  |
| B0BNF9 | *Hao1* | Hydroxyacid oxidase 1 | 0.0080^§^ | 1.60^§^ | ** |  |  |  |  |
| CBG | *Serpina6* | Corticosteroid-binding globulin | 0.0131^§^ | 1.51^§^ | * |  |  |  |  |
| D4A678 | *Spta1* | Spectrin, alpha, erythrocytic 1 | 0.0191^§^  0.0047^Δ^ | 1.43^§^  -1.11^Δ^ | * |  |  | ** |  |
| SDC4 | *Sdc4* | Syndecan-4 | 0.0284^§^  0.0243^$^  0.0461^#^ | 1.33^§^  1.28^$^  1.07^#^ | * | * | * |  |  |
| TSC1 | *Tsc1* | Hamartin | 0.0049^§^  0.0362^$^ | 1.21^§^  0.85^$^ | ** | * |  |  |  |
| CRP | *Crp* | C-reactive protein | 0.0053^§^  0.0010^Δ^ | 1.20^§^  -1.27^Δ^ | ** |  |  | ** |  |
| AFAM | *Afm* | Afamin | 0.0127^§^  0.0032^Δ^ | 1.18^§^  -1.22^Δ^ | * |  |  | ** |  |
| IGS11 | *Igsf11* | Immunoglobulin superfamily  member 11 | 0.0284^§^  0.0016^$^ | -1.64^§^  -1.92^$^ | * | ** |  |  |  |
| F1LWG4 | *Ndufaf1* | Complex I intermediate-associated protein 30, mitochondrial | 0.0188^§^  0.0097^Δ^ | -1.68^§^  1.78^Δ^ | * |  |  | ** |  |
| KDF1 | *Kdf1* | Keratinocyte differentiation factor 1 | 0.0012^§^  0.0249^$^  0.0065^Δ^ | -1.73^§^  -0.80^$^  1.38^Δ^ | ** | * |  | ** |  |
| PI5PA | *Inpp5j* | Phosphatidylinositol 4,5-bisphosphate 5-phosphatase A | 0.0210^§^  0.0045^$^  0.0390^†^ | -1.77^§^  -1.82^$^  -0.85^†^ | * | ** |  |  | * |
| D3ZSN7 | *Slc25a44* | Solute carrier family 25, member 44 | <0.0001^§^ | -2.07^§^ | *** |  |  |  |  |
| F1LYA6 | *Abi2* | Abl-interactor 2 | 0.0137^§^ | -2.11^§^ | * |  |  |  |  |
| AUP1 | *Aup1* | Lipid droplet-regulating VLDL assembly factor AUP1 | 0.0470^§^ | -2.16^§^ | * |  |  |  |  |
| PPR1A | *Ppp1r1a* | Protein phosphatase 1 regulatory subunit 1A | 0.0012^§^  <0.0001^$^ | -2.34^§^  -2.52^$^ | ** | *** |  |  |  |
| NECP1 | *Necap1* | Adaptin ear-binding coat-associated protein 1 | 0.0138^§^ | -2.45^§^ | * |  |  |  |  |
| D3ZAZ0 | *Eif3m* | Eukaryotic translation initiation factor 3 subunit M | 0.0023^§^  0.0324^Δ^ | -2.56^§^  2.06^Δ^ | ** |  |  | * |  |
| GPM6A | *Gpm6a* | Neuronal membrane glycoprotein M6-a | 0.0442^$^ | 1.78^$^ |  | * |  |  |  |
| B5DF86 | *Cog8* | Conserved oligomeric Golgi complex subunit 8 | 0.0226^$^ | 1.62^$^ |  | * |  |  |  |
| S47A1 | *Slc47a1* | Multidrug and toxin extrusion protein 1 | 0.0482^$^ | 1.33^$^ |  | * |  |  |  |
| Q6YDN8 | *Gid8* | GID complex subunit 8 homolog | 0.0045^$^ | 1.29^$^ |  | ** |  |  |  |
| ITB3 | *Itgb3* | **Integrin beta-3** | 0.0389^$^ | 1.23^$^ |  | * |  |  |  |
| A0A0G2JTY3; Q6IRK4 | *Slc37a4* | Solute carrier family 37 member 4 | 0.0308^$^  0.0389^#^ | 1.20^$^  1.23^#^ |  | * | * |  |  |
| G3PT | *Gapdhs* | Glyceraldehyde-3-phosphate dehydrogenase, testis-specific | 0.0283^$^ | 1.19^$^ |  | * |  |  |  |
| HMGN5 | *Hmgn5* | High mobility group nucleosome-binding domain-containing protein 5 | 0.0005^$^  0.0308^†^ | 1.07^$^  0.42^†^ |  | *** |  |  | * |
| A0A0G2K3E7; V9GZ80 | *LOC103690120; Nat8f1* | Camello-like 2, isoform CRA_a | 0.0070^$^ | 0.99^$^ |  | ** |  |  |  |
| A0A0G2KA91; E9PT68 | *Mier1* | Mesoderm induction early response protein 1 | 0.0131^$^ | 0.99^$^ |  | * |  |  |  |
| D3Z8L5 | *Pum1* | Pumilio RNA-binding family member 1 | 0.0107^$^ | -1.24^$^ |  | * |  |  |  |
| SPF30 | *Smndc1* | Survival of motor neuron-related-splicing factor 30 | 0.0194^$^ | -1.28^$^ |  | * |  |  |  |
| D3ZQC6 | *Ubr1* | E3 ubiquitin-protein ligase UBR4 | 0.0058^$^ | -1.29^$^ |  | ** |  |  |  |
| MAVS | *Mavs* | Mitochondrial antiviral-signaling protein | 0.0117^$^ | -1.30^$^ |  | * |  |  |  |
| PRDM2 | *Prdm2* | PR domain zinc finger protein 2 | 0.0011^$^ | -1.34^$^ |  | ** |  |  |  |
| MAP6 | *Map6* | Microtubule-associated protein 6 | 0.0076^$^ | -1.48^$^ |  | ** |  |  |  |
| CDC26 | *Cdc26* | Anaphase-promoting complex subunit CDC26 | <0.0001^$^ | -1.51^$^ |  | *** |  |  |  |
| Q5EB56 | *Mak16* | Protein MAK16 homolog | 0.0009^$^ | -1.52^$^ |  | *** |  |  |  |
| SYUG | *Sncg* | Gamma-synuclein | 0.0073^$^ | -1.67^$^ |  | ** |  |  |  |
| A0A0G2K926; D4AA52 | *LOC297568* | Murinoglobulin-1 | 0.0462^$^ | -3.41^$^ |  | * |  |  |  |
| HMCS2 | *Hmgcs2* | Hydroxymethylglutaryl-CoA synthase, mitochondrial | 0.0008^#^  <0.0001^Δ^  <0.0001^†^ | 3.10^#^  2.31^Δ^  4.46^†^ |  |  | *** | *** | *** |
| PSPB | *Sftpb* | Pulmonary surfactant-associated protein B | 0.0372^#^  0.0299^†^ | 1.69^#^  1.84^†^ |  |  | * |  | * |
| A0A0G2K8D1; D4A4X7 | *Mospd2* | Motile sperm domain-containing 2 | 0.0254^#^ | 1.30^#^ |  |  | * |  |  |
| LAT2 | *Slc7a8* | Large neutral amino acids transporter small subunit 2 | 0.0494^#^ | 1.27^#^ |  |  | * |  |  |
| PXMP4 | *Pxmp4* | Peroxisomal membrane protein 4 | 0.0017^#^  0.0034^†^ | 1.10^#^  1.12^†^ |  |  | ** |  | ** |
| Q4KLZ0 | *Vnn1* | Vanin 1 | 0.0008^#^  0.0033^Δ^  <0.0001^†^ | 1.07^#^  0.69^Δ^  1.20^†^ |  |  | *** | ** | *** |
| DHCR7 | *Dhcr7* | 7-dehydrocholesterol reductase | 0.0187^#^  0.0290^Δ^  0.0326^†^ | 1.00^#^  1.14^Δ^  0.90^†^ |  |  | * | * | * |
| MDR1 | *Abcb1* | ATP-dependent translocase ABCB1 | 0.0085^#^  0.0032^†^ | 0.83^#^  0.93^†^ |  |  | ** |  | ** |
| BACH | *Acot7* | Cytosolic acyl coenzyme A thioester hydrolase | <0.0001^#^  0.0004^Δ^  0.0001^†^ | 0.80^#^  0.82^Δ^  1.00^†^ |  |  | *** | *** | *** |
| D3ZE02 | *LOC100361913* | Immortalization up-regulated protein-like | 0.0469^#^ | 0.80^#^ |  |  | * |  |  |
| F1M4H5 | *Nova2* | NOVA alternative-splicing regulator 2 | 0.0378^#^ | -0.75^#^ |  |  | * |  |  |
| Q0R3X4; Q6IRE3 | *Gimap4* | GTPase IMAP family member 4 | 0.0262^#^ | -0.81^#^ |  |  | * |  |  |
| A0A0G2JZT5; D4A0F5 | *Sept7* | Septin | 0.0165^#^ | -0.87^#^ |  |  | * |  |  |
| Q5XIE4 | *Rchy1* | Ring finger and CHY zinc finger domain containing 1, isoform CRA_b | 0.0499^#^ | -0.88^#^ |  |  | * |  |  |
| TM252 | *Tmem252* | Transmembrane protein 252 | 0.0401^#^ | -1.05^#^ |  |  | * |  |  |
| GBG11 | *Gng11* | Guanine nucleotide-binding protein G(I)/G(S)/G(O) subunit gamma-11 | 0.0149^#^ | -1.25^#^ |  |  | * |  |  |
| UBP4 | *Usp4* | Ubiquitin carboxyl-terminal hydrolase 4 | 0.0422^#^ | -1.44^#^ |  |  | * |  |  |
| APLP2 | *Aplp2* | Amyloid-like protein 2 | 0.0236^#^ | -1.53^#^ |  |  | * |  |  |
| E9PU07 | *Ear1* | Eosinophil-associated, ribonuclease A family, member 1 | 0.0069^#^ | -1.65^#^ |  |  | ** |  |  |
| FABP4 | *Fabp4* | Fatty acid-binding protein, adipocyte | 0.0084^#^  0.0490^Δ^  0.0498^†^ | -2.68^#^  -1.45^Δ^  -0.59^†^ |  |  | ** | * | * |
| F1LZ81; M0R6K4 | *Dock4* | Dedicator of cytokinesis 4 | 0.0046^Δ^  0.0166^†^ | 3.67^Δ^  -3.59^†^ |  |  |  | ** | * |
| A8QIC3 | *Dnajc9* | DnaJ (Hsp40) homolog, subfamily C, member 9 (Predicted) | 0.0005^Δ^ | 2.33^Δ^ |  |  |  | *** |  |
| A0A0G2JY11; D4AAV9 | *Tspan9* | Tetraspanin | 0.0311^Δ^ | 1.48^Δ^ |  |  |  | * |  |
| D3ZDI7 | *Ppp2r5a* | Serine/threonine-protein phosphatase 2A 56 kDa regulatory subunit | 0.0126^Δ^ | 1.30^Δ^ |  |  |  | * |  |
| D3ZVI7 | *Sass6* | Spindle assembly abnormal protein 6 homolog | 0.0064^Δ^ | 1.26^Δ^ |  |  |  | ** |  |
| A0A0G2JUI4; A0A0G2K602 | *Noa1* | Nitric oxide-associated 1 | 0.0192^Δ^ | 1.26^Δ^ |  |  |  | * |  |
| D4ABI7 | *Hacd3* | Very-long-chain (3R)-3-hydroxyacyl-CoA dehydratase | 0.0187^Δ^ | 1.20^Δ^ |  |  |  | * |  |
| TM2D2 | *Tm2d2* | TM2 domain-containing protein 2 | 0.0094^Δ^ | 1.19^Δ^ |  |  |  | ** |  |
| COG6 | *Cog6* | Conserved oligomeric Golgi complex subunit 6 | 0.0452^Δ^ | 1.04^Δ^ |  |  |  | * |  |
| RUSD4 | *Rpusd4* | Mitochondrial RNA pseudouridine synthase Rpusd4 | 0.0271^Δ^ | 1.02^Δ^ |  |  |  | * |  |
| A0A0G2K855; D3ZT95 | *Ercc4* | ERCC4 domain-containing protein | 0.0217^Δ^ | -1.00^Δ^ |  |  |  | * |  |
| SMAD4 | *Smad4* | Mothers against decapentaplegic homolog 4 | 0.0282^Δ^ | -1.02^Δ^ |  |  |  | * |  |
| M0R4I7 | *Atp23* | Mitochondrial inner membrane protease ATP23 | 0.0491^Δ^ | -1.06^Δ^ |  |  |  | * |  |
| M0R485 | *Pglyrp2* | Peptidoglycan recognition protein 2 | 0.0092^Δ^ | -1.19^Δ^ |  |  |  | ** |  |
| UBP46 | *Usp46* | Ubiquitin carboxyl-terminal hydrolase 46 | 0.0005^Δ^ | -1.33^Δ^ |  |  |  | *** |  |
| Q62669 | *LOC103694855* | Globin a1 | 0.0152^Δ^ | -1.38^Δ^ |  |  |  | * |  |
| D4AAE6 | *Rab20* | RAB20, member RAS oncogene family | 0.0283^Δ^ | -1.38^Δ^ |  |  |  | * |  |
| CAH1 | *Ca1* | Carbonic anhydrase 1 | 0.0071^Δ^ | -1.46^Δ^ |  |  |  | ** |  |
| SPA3M | *Serpina3m* | Serine protease inhibitor A3M | 0.0341^Δ^ | -1.46^Δ^ |  |  |  | * |  |
| D3ZW09 | *Sorcs2* | Sortilin-related VPS10 domain-containing receptor 2 | 0.0037^Δ^ | -1.56^Δ^ |  |  |  | ** |  |
| ZKSC1 | *Zkscan1* | Zinc finger protein with KRAB and SCAN domains 1 | 0.0047^†^ | 1.55^†^ |  |  |  |  | ** |
| A3KNA0 | *Aqr* | RNA helicase aquarius | 0.0194^†^ | 1.34^†^ |  |  |  |  | * |
| GUC2B | *Guca2b* | Guanylate cyclase activator 2B | 0.0323^†^ | 1.12^†^ |  |  |  |  | * |
| KEG1 | *Keg1* | Glycine N-acyltransferase-like protein Keg1 | 0.0030^†^ | 0.97^†^ |  |  |  |  | ** |
| Q6TXF6 | *Decr2* | NME/NM23 nucleoside diphosphate kinase 4 | 0.0136^†^ | 0.96^†^ |  |  |  |  | * |
| A0A1W2Q5Z6 | *Tax1bp3* | Tax1-binding protein 3 | 0.0326^†^ | 0.89^†^ |  |  |  |  | * |
| TIP | *Itfg1* | T-cell immunomodulatory protein | 0.0250^†^ | 0.87^†^ |  |  |  |  | * |
| GSTA2 | *Gsta2* | Glutathione S-transferase alpha-2 | 0.0477^†^ | 0.86^†^ |  |  |  |  | * |
| AQP3 | *Aqp3* | Aquaporin-3 | 0.0086^†^ | 0.80^†^ |  |  |  |  | ** |
| B1H269 | *Ddx27* | RNA helicase | 0.0350^†^ | 0.79^†^ |  |  |  |  | * |
| B5DFK2 | *Sash3* | SAM and SH3 domain-containing 3 | 0.0218^†^ | -0.80^†^ |  |  |  |  | * |
| CLC11 | *Clec11a* | C-type lectin domain family 11 member A | 0.0122^†^ | -0.86^†^ |  |  |  |  | * |
| D3ZBC7 | *Dhrs13* | Dehydrogenase/reductase 13 | 0.0047^†^ | -0.92^†^ |  |  |  |  | ** |
| MYO9A | *Myo9a* | Unconventional myosin-Ixa | 0.0264^†^ | -0.96^†^ |  |  |  |  | * |
| NKAP | *Nkap* | NF-kappa-B-activating protein | 0.0477^†^ | -1.09^†^ |  |  |  |  | * |
| B4F7B2 | *Taf10* | Transcription initiation factor TFIID subunit 10 | 0.0258^†^ | -1.22^†^ |  |  |  |  | * |
| Q3B7U1 | *Maged2* | MAGE family member D2 | 0.0481^†^ | -1.27^†^ |  |  |  |  | * |
| D3ZA66 | *Pstpip2* | Proline-serine-threonine phosphatase-interacting protein 2 | 0.0065^†^ | -1.29^†^ |  |  |  |  | ** |
| FRMD8 | *Frmd8* | FERM domain-containing protein 8 | 0.0316^†^ | -1.49^†^ |  |  |  |  | * |
| A1BG | *A1bg* | Alpha-1B-glycoprotein | 0.0061^†^ | -2.74^†^ |  |  |  |  | ** |

Proteome data from n= 6 female rats per group. Protein, gene, and protein description are in accordance with the UniProt database. Statistical analysis was performed by a one-way ANNOVA and a two-sample t-test, p< 0.05 was considered significant. Proteins with a log2 fold change (fc) of > |0.58| (≙ fc> 1.5) were considered relevantly altered. P-values and the log2 fc were included for all significant group differences. Color mapping represents the fc of group differences in protein expression (upregulated proteins “red” downregulated proteins “blue”). The following group differences were analyzed: 1) Model effect: §, LIG-CONTR vs. C-CONTR; and $, IUS-CONTR vs. C-CONTR. 2) Diet effect: #, C-N3PUFA vs. C-CONTR; Δ, LIG-N3PUFA vs. LIG-CONTR; †, IUS-N3PUFA vs. IUS-CONTR.


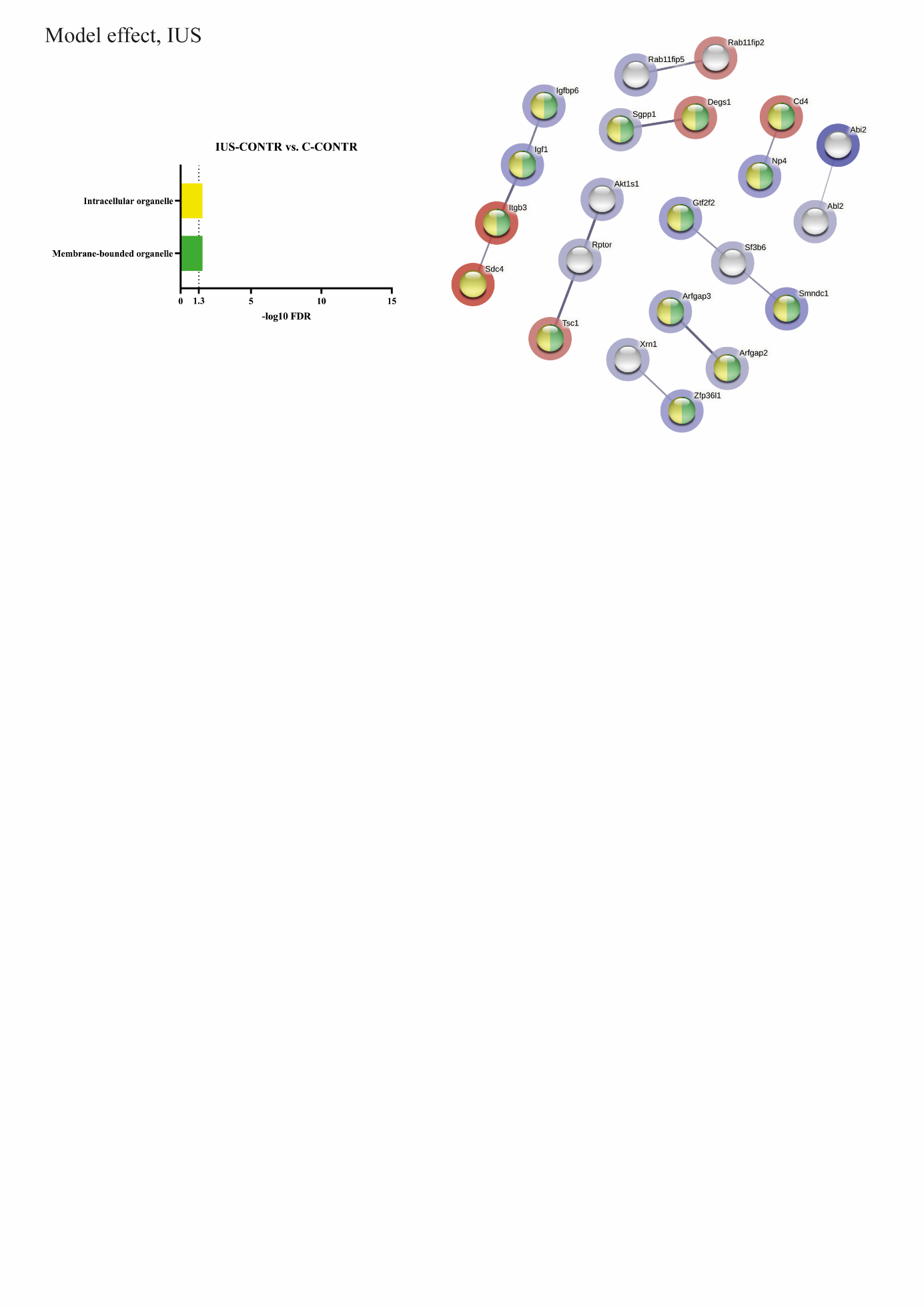


**Supplemental Figure 1:** String analysis of relevantly altered kidney cortex(p< 0.05, fc>1.5) kidney cortex proteins on P39 between IUS-CONTR and C-CONTR. Colored nodes represent KEGG pathways or GO terms (Membrane-bounded organelle, GO:0043227; intracellular organelle, GO:0043229) within a –log10 false discovery rate (FDR) of > 1.3.
